# Class-II dihydroorotate dehydrogenases from three phylogenetically distant fungi support anaerobic pyrimidine biosynthesis

**DOI:** 10.1186/s40694-021-00117-4

**Published:** 2021-10-16

**Authors:** Jonna Bouwknegt, Charlotte C. Koster, Aurin M. Vos, Raúl A. Ortiz-Merino, Mats Wassink, Marijke A. H. Luttik, Marcel van den Broek, Peter L. Hagedoorn, Jack T. Pronk

**Affiliations:** 1 Department of Biotechnology, Delft University of Technology, van der Maasweg 9, 2629 HZ Delft, The Netherlands; 2 Wageningen Plant Research, Wageningen University and Research, Droevendaalsesteeg 1, 6708 PB Wageningen, The Netherlands

**Keywords:** Yeast, Neocallimastigomycota, Oxygen, Anaerobiosis, Oxygen requirements, Uracil

## Abstract

**Background:**

In most fungi, quinone-dependent Class-II dihydroorotate dehydrogenases (DHODs) are essential for pyrimidine biosynthesis. Coupling of these Class-II DHODHs to mitochondrial respiration makes their in vivo activity dependent on oxygen availability. *Saccharomyces cerevisiae* and closely related yeast species harbor a cytosolic Class-I DHOD (Ura1) that uses fumarate as electron acceptor and thereby enables anaerobic pyrimidine synthesis. Here, we investigate DHODs from three fungi (the Neocallimastigomycete *Anaeromyces robustus* and the yeasts *Schizosaccharomyces japonicus* and *Dekkera bruxellensis*) that can grow anaerobically but, based on genome analysis, only harbor a Class-II DHOD.

**Results:**

Heterologous expression of putative Class-II DHOD-encoding genes from fungi capable of anaerobic, pyrimidine-prototrophic growth (*Arura9, SjURA9, DbURA9*) in an *S. cerevisiae ura1Δ* strain supported aerobic as well as anaerobic pyrimidine prototrophy. A strain expressing *DbURA9* showed delayed anaerobic growth without pyrimidine supplementation. Adapted faster growing *DbURA9*-expressing strains showed mutations in *FUM1*, which encodes fumarase. GFP-tagged SjUra9 and DbUra9 were localized to *S. cerevisiae* mitochondria, while ArUra9, whose sequence lacked a mitochondrial targeting sequence, was localized to the yeast cytosol. Experiments with cell extracts showed that ArUra9 used free FAD and FMN as electron acceptors. Expression of *SjURA9* in *S. cerevisiae* reproducibly led to loss of respiratory competence and mitochondrial DNA. A cysteine residue (C265 in SjUra9) in the active sites of all three anaerobically active Ura9 orthologs was shown to be essential for anaerobic activity of SjUra9 but not of ArUra9.

**Conclusions:**

Activity of fungal Class-II DHODs was long thought to be dependent on an active respiratory chain, which in most fungi requires the presence of oxygen. By heterologous expression experiments in *S. cerevisiae*, this study shows that phylogenetically distant fungi independently evolved Class-II dihydroorotate dehydrogenases that enable anaerobic pyrimidine biosynthesis. Further structure–function studies are required to understand the mechanistic basis for the anaerobic activity of Class-II DHODs and an observed loss of respiratory competence in *S. cerevisiae* strains expressing an anaerobically active DHOD from *Sch. japonicus*.

**Supplementary Information:**

The online version contains supplementary material available at 10.1186/s40694-021-00117-4.

## Background

Oxidation of dihydroorotate to orotate, an essential reaction for pyrimidine biosynthesis in all domains of life, is catalyzed by the flavoprotein dihydroorotate dehydrogenase (DHOD) [1, 2]. Depending on the type of DHOD, different electron acceptors are used for re-oxidation of the flavin cofactor. Soluble Class-I DHODs include homodimeric fumarate-dependent type I-A enzymes and heterotetrameric NAD^+^-dependent type I-B enzymes [3, 4], while membrane-bound Class-II DHODs are quinone-dependent and coupled to respiration [5]. Protein sequence identity between Class-I and Class-II DHODs is only approximately 20% [6–8].

Class I-A and Class-II DHODs contain a single FMN cofactor per subunit. In heterotetrameric Class I-B enzymes, two subunits also each contain an FMN cofactor, while the other two both contain an FAD cofactor and an [2Fe-2S] cluster [4]. Class-I DHODs contain an active-site cysteine residue involved in deprotonation of C5 of dihydroorotate, while Class-II enzymes have a serine in the same position acting as the catalytic base [9–13].

Most eukaryotes harbor a monomeric Class-II DHOD that donates electrons to the quinone pool of the mitochondrial respiratory chain [8, 14–16]. Bacterial Class-II DHODs have an N-terminal sequence that localizes them to the inside of the cytoplasmic membrane, whereas eukaryotic enzymes are targeted to the outside of the mitochondrial inner membrane [5]. This mitochondrial targeting also applies to fungal Class-II DHODs, which include the Ura9 orthologs of yeasts such as *Lachancea kluyveri* and *Schizosaccharomyces pombe* [17]. Since respiration in yeasts requires oxygen as electron acceptor, reliance on these respiration-coupled enzymes precludes pyrimidine-prototrophic anaerobic growth [8].

Class-I DHODs predominantly occur in gram-positive bacteria and Archaea [18] but are also found in a small number of yeasts, including *Saccharomyces cerevisiae* and closely related species [19, 20]. *S. cerevisiae* is among the few yeast species that are able to grow under strictly anaerobic conditions [21]. ScUra1, a Class-IA, fumarate-coupled DHOD, enables *S. cerevisiae* to synthesize pyrimidines in the absence of oxygen [19, 20]. A small number of other Saccharomycetes, including *Kluyveromyces lactis* and *L. kluyveri*, harbor Ura1 as well as Ura9 orthologs [19, 22, 23]. Based on sequence similarity of yeast *ScURA1* orthologs with *Lactococcus* genes, they are assumed to have been acquired by horizontal gene transfer [15, 19].

In line with a proposed essentiality of *ScURA1* orthologs for anaerobic pyrimidine synthesis by yeasts [19, 24], replacement of *ScURA1* by a Class-II DHOD gene from *L. kluyveri (LkURA9)* or *Sch. pombe* (*SpURA3*) yielded strains that were only pyrimidine prototrophic under aerobic conditions [8, 19]. Conversely, replacement of *ScURA1* by *LkURA1* supported aerobic as well as anaerobic pyrimidine prototrophy [19]. Introduction of *ScURA1* in *URA9*-dependent yeasts was proposed as a metabolic engineering strategy for enabling anaerobic, pyrimidine-prototrophic growth of yeasts lacking a native *ScURA1* ortholog [25].

The long-held assumption that expression of a Class-I DHOD is required for anaerobic pyrimidine biosynthesis in eukaryotes was first challenged when *Dekkera bruxellensis*, which only harbors a putative *URA9* ortholog, was shown to grow anaerobically in pyrimidine-free media [26, 27]. A hypothesis that DbUra9 is able to use a non-quinone electron acceptor [17, 28, 29] was, however, not experimentally tested.

We recently observed that *D. bruxellensis* may not be the only eukaryote in which anaerobic pyrimidine synthesis involves a Class-II DHOD. Inspection of the genome of the fission yeast *Sch. japonicus,* which shows fast anaerobic growth in synthetic media without uracil [30, 31], suggested that it only contains a *URA9* ortholog. Moreover, genomes of Neocallimastigomycetes, a group of deep-branching, obligately anaerobic fungi that lack mitochondria and instead harbor hydrogenosomes [32, 33], also appeared to lack orthologs of soluble Class-I DHOD.

The goals of the present study were to investigate whether *URA9* orthologs in eukaryotes capable of anaerobic growth indeed support anaerobic pyrimidine biosynthesis, and to gain more insight into underlying mechanisms and trade-offs. To this end, we expressed putative Class-II DHOD genes from the obligately anaerobic Neocallimastigomycete *Anaeromyces robustus* (*Arura9*), the facultative anaerobes *Sch. japonicus* (*SjURA9*) and *D. bruxellensis* (*DbURA9*), as well as from the oxygen-requiring yeasts *Ogataea parapolymorpha* (*OpURA9*) and *Kluyveromyces marxianus* (*KmURA9*), in an *S. cerevisiae ura1Δ* background. After studying aerobic and anaerobic growth of the resulting strains in uracil-supplemented and uracil-free synthetic media, we analyzed subcellular localization of Ura9-eGFP fusion proteins in *S. cerevisiae* and assessed the impact of a conserved amino-acid substitution in anaerobically functional Ura9 orthologs. To identify possible natural electron acceptors, we performed enzyme assays in cell extracts of an *S. cerevisiae* strain expressing ArUra9 and re-sequenced the genomes of laboratory-evolved *S. cerevisiae* strains whose anaerobic growth depended on expression of *DbURA9*. We found that instead of quinone, ArUra9 uses free flavins as electron acceptors and that expression of *SjURA9* in *S. cerevisiae* results in loss of respiration.

## Results

### Obligately anaerobic Neocallimastigomycetes and facultatively anaerobic yeasts harbor putative Class-II DHODs

A preliminary exploration of a set of selected fungal proteomes for Class-I and Class-II DHODs was based on a sequence similarity search with Class-I and Class-II enzymes of *L. kluyveri* (LkUra1 and LkUra9, respectively; [19]; Table 1) as queries. Consistent with earlier studies, the *S. cerevisiae* proteome showed a single sequence with strong similarity to LkUra1 (ScUra1; [10, 19, 23]), while proteomes of the facultatively fermentative yeasts *O. parapolymorpha* and *K. marxianus*, which both require oxygen for biosynthesis-related reactions [34, 35]**,** yielded previously described Class-II DHOD sequences with strong homology to LkUra9 (OpUra9 and KmUra9, respectively; [23, 36]). *K. marxianus* additionally showed a sequence with high sequence similarity to LkUra1 (KmUra1; [22]). The search also confirmed earlier reports that the facultatively anaerobic yeast *D. bruxellensis* only harbors a putative DHOD sequence with high similarity to LkUra9 (DbUra9; [17, 23, 28]).

**Table 1:** *L. kluyveri* LkUra1 and LkUra9 sequence homology results using selected fungal proteomes

Subject proteome	Resulting GenBank accession	Query coverage (%)		E-value		Identity (%)		Interpretation	
		LkUra1	LkUra9	LkUra1	LkUra9	LkUra1	LkUra9	Ura1	Ura9
*D. bruxellensis* [91]	XP_041139490.1	95	90	5 ⋅ 10^–17^	4 ⋅ 10^–145^	24.5	50.9	No	Yes
*A. robustus* [108, 109]	ORX87218.1	56	77	3 ⋅ 10^–9^	2 ⋅ 10^–85^	27.0	44.6	No	Yes
*P. finnis* [108, 109]	ORX52621.1	56	77	5 ⋅ 10^–9^	7 ⋅ 10^–86^	24.9	44.9	No	Yes
*N. californiae* [108]	ORY72481.1	54	74	2 ⋅ 10^–8^	5 ⋅ 10^–82^	27.7	44.9	No	Yes
*Sch. japonicus* [110]	XP_002171492.1	63	97	2 ⋅ 10^–13^	1 ⋅ 10^–113^	30.6	44.3	No	Yes
*S. cerevisiae* [111]	NP_012706.1	100	71	0.0	4 ⋅ 10^–9^	80.3	23.8	Yes	No
*K. marxianus* [112]	XP_022674337.1	100	73	0.0	2 ⋅ 10^–12^	77.1	25.1	Yes	No
	XP_022675611.1	94	95	6 ⋅ 10^–11^	0.0	24.0	73.7	No	Yes
*O. parapolymorpha* [113]	XP_013936870.1	95	91	1 ⋅ 10^–9^	0.0	23.0	63.4	No	Yes

Proteomes of the Neocallimastigomycetes *A. robustus* (NCBI taxid 1754192), *P. finnis* (1754191), *N. californiae* (1754190), and the yeasts *Sch. japonicus* (402676), *D. bruxellensis* (5007), *K. marxianus* (1003335), *O. parapolymorpha* (871575) and *S. cerevisiae* (559292) were subjected to blastp searches using LkUra1 (DHOD Class I-A, UniProt KB accession number Q7Z892) and LkUra9 (DHOD Class II, accession number Q6V3W9) amino acid sequences as queries

Sequence comparison with LkUra1 provided no evidence for presence of a Class-I DHOD in the Neocallimastigomycetes *Piromyces finnis, Neocallimastix californiae* and *A. robustus*. Instead, these obligately anaerobic fungi yielded predicted protein sequences with high similarity to the Class-II DHOD LkUra9 (Table 1), which we tentatively called PfUra9, NcUra9 and ArUra9, respectively. A similar result was obtained for the facultatively anaerobic fission yeast *Sch. japonicus* [30, 31], whose putative Class-II DHOD was tentatively named SjUra9 (Table 1). Dependence of anaerobic, pyrimidine-prototrophic growth of phylogenetically distant fungi on Class-II (‘Ura9’) DHODs would be remarkable in view of the well-documented coupling of canonical eukaryotic Class-II DHODs to aerobic mitochondrial respiration [5, 8, 19, 37, 38].

**Fig. 1:**
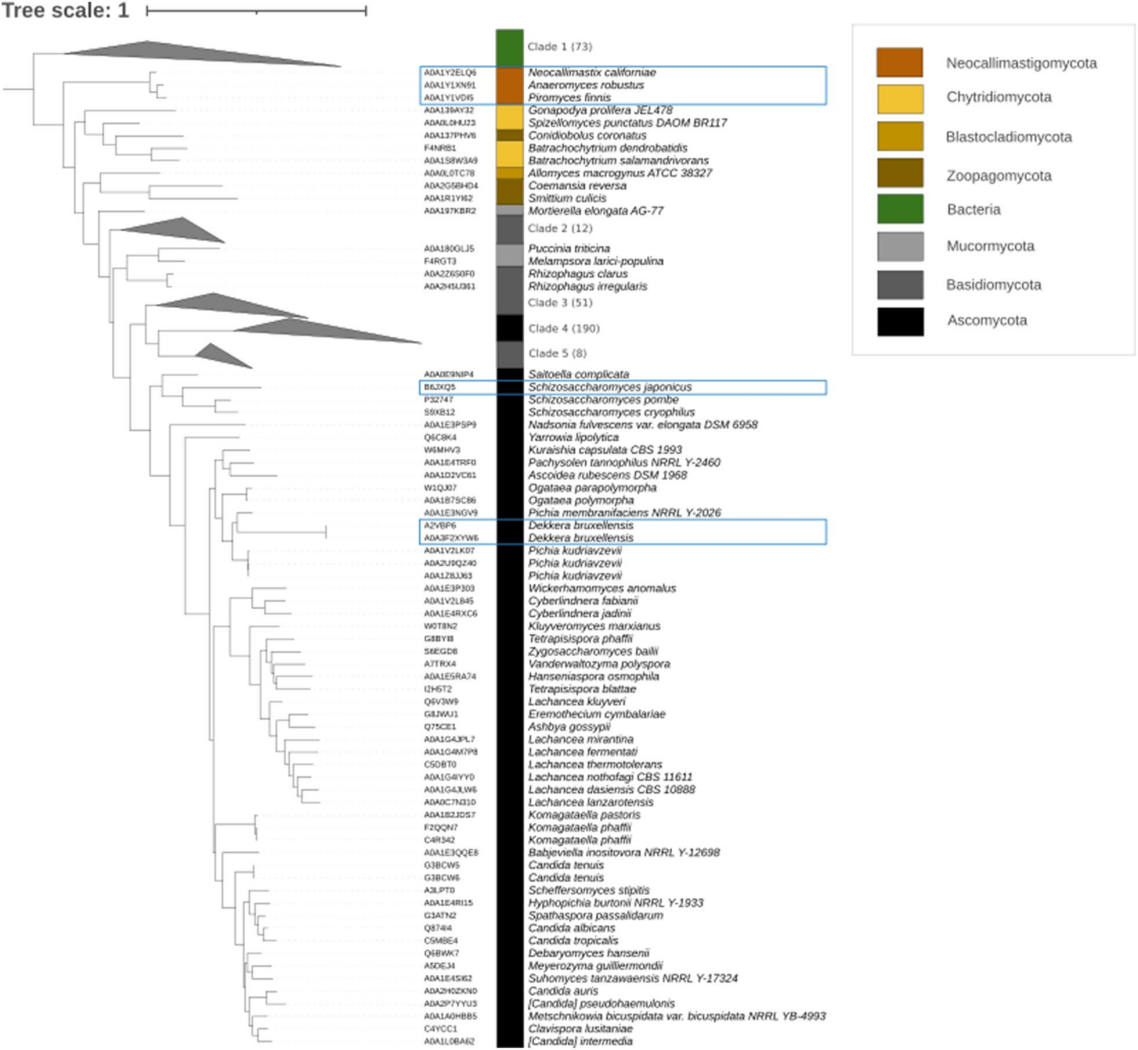
Phylogenetic tree of fungal Ura9 orthologs. An orthology search using *L. kluyveri* Class-II dihydroorotate dehydrogenase (LkUra9; UniProt KB accession number: Q6V3W9) as query yielded 331 fungal and 73 bacterial Ura9 orthologs (Additional file 1). These Ura9 orthologs were used to calculate a maximum-likelihood phylogenetic tree which was rooted using the bacterial clade as outgroup, and for which only bootstrap values above 75 are shown. Blue boxes indicate species capable of pyrimidine-prototrophic anaerobic growth. Numbers of sequences in collapsed clades are indicated and include Bacteria (clade 1), Basidiomycota (clade 2, 3 and 5) and Ascomycota (clade 4). The raw phylogenetic tree in phyml format is provided in Additional file 3, and interactive visualization is provided in iTOL (https://itol.embl.de/export/19319025314544511632749060)

To study the phylogeny of fungal Ura9 orthologs, the amino-acid sequence of LkUra9 was used as query for a sequence analysis with a hidden Markov model method (HMMER; [39]) against all fungal proteomes in Uniprot [40], from which the best hits were used for orthology prediction (see “Methods”). A similar strategy was performed to obtain bacterial LkUra9 orthologs. A phylogenetic tree constructed based on the resulting 331 fungal and 73 bacterial Ura9 orthologs showed a clear separation of bacterial and fungal Ura9 orthologs (Fig. 1; Additional file 1, Additional file 2, Additional file 3). The topology of the tree followed fungal phylogeny, as exemplified by the LkUra9 ortholog of *Sch. japonicus*, which clustered with that of closely related species *Sch. pombe* whose Class-II DHOD was previously shown not to support anaerobic uracil prototrophy [8]. Similarly, the *D. bruxellensis* Ura9 ortholog clustered with those of other Pichiacaea yeasts, including *O. parapolymorpha* whose Ura9 ortholog does not support anaerobic uracil prototrophy (see below). These results indicated that, if Ura9 orthologs of Neocallimastigomycetes, *D. bruxellensis* and *Sch. japonicus* share properties that enable anaerobic pyrimidine synthesis, these are likely to have evolved independently, without involvement of horizontal gene transfer (HGT). Evolution of oxygen-independent pyrimidine synthesis in these fungi would then differ from the proposed HGT-mediated acquisition of a respiration-independent Class-I DHOD by an ancestor of *S. cerevisiae* [19, 20] and from proposed HGT-driven adaptations of *Sch. japonicus* [31, 41] and Neocallimastigomycetes [42, 43] to bypass other oxygen requirements for biosynthetic processes.

### Heterologous *URA9* genes complement aerobic pyrimidine auxotrophy of *ura1Δ S. cerevisiae*

To assess functionality of the putative Class-II DHOD genes of the Neocallimastigomycete *A. robustus* and of the facultatively anaerobic yeasts *Sch. japonicus* and *D. bruxellensis*, the *ScURA1* open-reading frame of *S. cerevisiae* was replaced by expression cassettes for codon-optimized *Arura9*, *SjURA9* or *DbURA9* coding sequences (Additional file 4). In addition, strains were constructed in which *ScURA1* was replaced by expression cassettes for *URA9* orthologs of the aerobic yeasts *K. marxianus* and *O. parapolymorpha*.

**Fig. 2:**
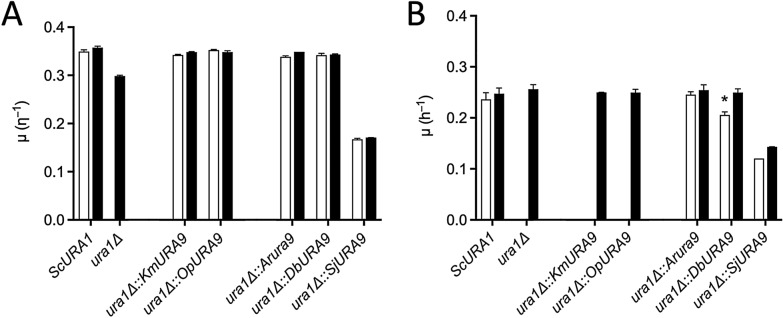
Complementation of uracil auxotrophy of *S. cerevisiae ura1Δ* strains by expression of heterologous *URA9* orthologs. The native *ScURA1* gene of *S. cerevisiae* was replaced by *URA9* orthologs from *K. marxianus* (Km), *O. parapolymorpha* (Op), *A. robustus* (Ar), *D. bruxellensis* (Db) or *Sch. japonicus* (Sj). **A** Aerobic cultures, **B**: anaerobic cultures. Open bars indicate specific growth rates (μ) on synthetic medium without uracil (SMUD), closed bars indicate μ in uracil-supplemented synthetic medium (SMUD + ura). Relevant genotypes of *S. cerevisiae* strains: CEN.PK113-7D, *ScURA1* reference strain; IMK824, *ura1Δ* reference strain; IMI446, *ura1Δ*::*KmURA9*; IMI447, *ura1Δ*::*OpURA9*; IMI432, *ura1Δ*::*Arura9*; IMI439, *ura1Δ*::*DbURA9*; IMI452, *ura1Δ*::*SjURA9*. *Since strain IMI439 (*ura1Δ*::*DbURA9*) showed delayed growth in initial anaerobic cultures on SMUD, its specific growth rate on SMUD was measured after transfer to a second culture. Data represent the average from biological duplicates and mean deviation (Additional file 5; Table S1)

The *S. cerevisiae* reference strain CEN.PK113-7D (*URA1*) grew fast (0.36 h^−1^) in anaerobic cultures on glucose-containing synthetic media with and without uracil (SMUD + ura and SMUD, respectively), while the congenic *ura1Δ* strain IMK824 only grew on SMUD + ura (0.30 h^−1^, Fig. 2, Additional file 5: Table S1). The lower specific growth rate of the uracil-auxotrophic strain IMK824 on SMUD + ura probably reflected a growth-limiting rate of uracil uptake [44]. *S. cerevisiae ura1Δ* strains carrying expression cassettes for *Arura9*, *DbURA9*, *KmURA9* or *OpURA9* all grew aerobically on SMUD + ura and SMUD, at specific growth rates of 0.34 h^−1^ to 0.35 h^−1^. A *ura1Δ* strain expressing *SjURA9* grew almost two-fold slower (0.17–0.19 h^−1^) on SMUD than the other uracil-prototrophic strains (Fig. 2, Additional file 5: Table S1). The growth rate of the *SjURA9*-expressing strain on SMUD + ura also was approximately one-third lower than that of the *ura1Δ* strain IMK824, indicating that expression of *SjURA9* negatively affected aerobic growth of *S. cerevisiae* on glucose.

These results showed that ArUra9, SjUra9 and DbUra9 are functional DHODs that complement a *ura1Δ* mutation in *S. cerevisiae* under aerobic conditions*.* A similar complementation of *S. cerevisiae ura1* null mutants was previously demonstrated for Class-II DHODs of the oxygen-requiring yeasts *L. kluyveri* (LkUra9 [19]) and *Sch. pombe* (SpUra3 [8]).

### Class II-DHODs of *A. robustus*, *D. bruxellensis* and *Sch. japonicus* support anaerobic growth of an *S. cerevisiae ura1Δ* strain

*A. robustus*, *D. bruxellensis* and *Sch. japonicus* were previously reported to grow anaerobically in synthetic media without pyrimidine supplementation [26, 27, 30, 45]. While, based on genome-sequence analysis, previous studies [17, 23, 28, 29] inferred that anaerobic pyrimidine prototrophy of *D. bruxellensis* was supported by a Class-II DHOD (DbUra9), its activity was not experimentally confirmed. To assess anaerobic functionality of ArUra9, DbUra9 and SjUra9, we investigated whether these Class-II DHODs could support anaerobic, pyrimidine-prototrophic growth of *ura1Δ S. cerevisiae* strains.

As anticipated, the reference strain *S. cerevisiae* CEN.PK113-7D, which expresses the native fumarate-dependent Class I-A DHOD ScUra1, showed similar specific growth rates in anaerobic cultures on SMUD and SMUD + ura (0.24–0.25 h^−1^, Fig. 2, Additional file 5: Table S1), while strain IMK824 (*ura1Δ*) only grew anaerobically (0.26 h^−1^) on SMUD + ura (Fig. 2, Additional file 5: Table S1). Strains IMI446 and IMI447, in which *ScURA1* was replaced by *URA9* genes of the aerobic yeasts *K. marxianus* and *O. parapolymorpha*, only grew anaerobically when media were supplemented with uracil (Fig. 2, Additional file 5: Table S1). These results are in line with the coupling of canonical eukaryotic Class-II DHODs to the quinone pool of the mitochondrial respiratory chain [5, 8, 19, 37, 38] and with previous studies by Gojković et al*.* [19] and Nagy et al*.* [8].

In contrast to expression of *URA9* orthologs from oxygen-requiring yeasts, expression of *Arura9* in *S. cerevisiae* supported fast anaerobic growth (0.25 h^−1^) on SMUD (Fig. 2, Additional file 5: Table S1). An *S. cerevisiae* strain in which *ScURA1* was replaced by a *SjURA9* expression cassette also grew anaerobically on SMUD as well as SMUD + ura, at an approximately two-fold lower growth rate than that of the strain expressing *Arura9* (Fig. 2, Additional file 5: Table S1).

Strain IMI439, in which *DbURA9* replaced *ScURA1*, did not show anaerobic growth on SMUD during the first 30 h of incubation (Additional file 5: Figure S1). When, after 68 h, OD_600_ had increased to 2.9, the strain was transferred to fresh SMUD, resulting in immediate anaerobic growth. In a parallel experiment with strain IMI447, which expressed *KmURA9*, no growth was observed upon transfer to fresh SMUD.

These results indicated that Class-II DHODs from the Neocallimastigomycete *A. robustus* and from the facultatively anaerobic yeast *Sch. japonicus* support pyrimidine synthesis under anaerobic conditions. The delayed anaerobic growth of a *ura1Δ* strain expressing *DbURA9* suggested that anaerobic functionality of *DbURA9* in *S. cerevisiae* requires physiological or genetic adaptations.

### Fast anaerobic, uracil-prototrophic growth of DbUra9-dependent *S. cerevisiae* strains correlates with mutations in *FUM1*

To further explore anaerobic functionality of DbUra9, we investigated whether adaptation of *S. cerevisiae* IMI439 (*ura1Δ*::*DbURA9*) to anaerobic, pyrimidine-prototrophic growth (Additional file 5; Figure S1) was associated with mutations in the *DbURA9* expression cassette or in native *S. cerevisiae* genes. Two independent anaerobic cultures of this strain on SMUD were incubated until growth occurred and then transferred to fresh anaerobic SMUD, in which they instantaneously grew (Additional file 5: Figure S1). Upon reaching stationary phase, two single colonies were isolated from each culture. To check whether these isolates (IMS1167, IMS1168, IMS1169 and IMS1170) had acquired stable mutations that stimulated anaerobic, uracil-prototrophic growth, they were first grown under non-selective conditions (aerobic growth on SMUD + ura) and then transferred to anaerobic medium with a reduced uracil content (SMUD + ura0.1). Inoculation of anaerobic cultures on SMUD with the resulting anaerobic, pyrimidine-limited cultures resulted in instantaneous anaerobic growth of all four isolates (Additional file 5: Table S1). Comparison of whole-genome sequences of the four isolates with that of their parental strain IMI439 (*ura1Δ*::*DbURA9*) revealed no mutations in the *DbURA9* expression cassette. Strains IMS1167 and IMS1168, which originated from the same anaerobically adapted culture, both contained point mutations in *VPS1* (*VPS1*^I410L^), which encodes a dynamin-like GTPase involved in vacuolar sorting and in *FUM1* (*FUM1*^M432I^), encoding fumarase (Table 2). Strains IMS1169 and IMS1170, which were isolated from the second anaerobically adapted culture, each harbored a different mutation in *FUM1* (*FUM1*^*A294V*^ and *FUM1*^T218M^, respectively; Table 2). These results indicated that anaerobic activity of DbUra9 in *S. cerevisiae* does not require changes in its protein sequence but, based on the presence of mutations in *FUM1* in all four isolates, may depend on the intracellular fumarate concentration.

**Table 2:** Mutations in *DbURA9* expressing strains, evolved for anaerobic pyrimidine prototrophy

Evolution line	Strain	Mutations	
		Vps1	Fum1
Flask 1	IMS1167	I410L	M432I
	IMS1168	I410L	M432I
Flask 2	IMS1169	–	A294V
	IMS1170	–	I218M

Two cultures of IMI439 (*ura1Δ::DbURA9*) were independently evolved under anaerobic conditions on SMUD (Additional file 5: Figure S1). Two single colony isolates from each flask were subjected to whole-genome resequencing and predicted amino-acid substitutions were only found in Vps1 and Fum1

### A cysteine residue in the active site of SjUra9 is required for activity under anaerobic conditions

To identify potentially relevant differences in the amino-acid sequences of Ura9 orthologs from oxygen-requiring yeast strains and those of anaerobically functioning Ura9 enzymes, sequences of ArUra9, SjUra9, DbUra9, KmUra9 and OpUra9 were subjected to a multiple sequence alignment, along with those of the characterized Class II DHODs of *L. kluyveri* (LkUra9l), *Sch. pombe* (SpUra3), and DHOD sequences of the Neocallimastigomycetes *N. californiae* (NcUra9) and *P. finnis* (PfUra9) (Additional file 5: Figure S2). In comparison with the yeast Ura9 sequences, those of the three Neocallimastigomycetes showed a 76–81 amino-acid truncation N-terminal truncation. In canonical fungal Ura9 enzymes, the N-terminus contains a mitochondrial targeting sequence [5, 38] and is proposed to be involved in quinone binding [46, 47].

The Neocallimastigomycete Ura9 sequences as well as those of the two facultatively anaerobic yeasts (DbUra9 and SjUra9) contained a cysteine residue instead of the conserved serine residue that acts as catalytic base in canonical Class-II DHODs [11, 12, 48]. Of 331 fungal Ura9 orthologs (Additional file 1) only three additional proteins (from *Coemansia reversa*, *Smittium culisis* and *Gonapodya prolifera*) harbored a cysteine at this position, but did not show an N-terminal truncation (Additional file 1 and Additional file 5: Figure S2).

Since soluble fumarate- and NAD^+^-dependent Class-I DHODs also use a cysteine residue as catalytic base [12, 13], we investigated the relevance this residue for in vivo activity of ArUra9 and SjUra9 in *S. cerevisiae*. Because of the slow adaptation to anaerobic, uracil-prototrophic growth of IMI439 (*ura1Δ*::*DbURA9*), we did not include DbUra9 in these experiments. Point mutations were introduced in *Arura9* (C168S) and *SjURA9* (C265S) to change the cysteine codon for a serine, yielding strains IMG007 (*ura1Δ*::*Arura9*^C168S^) and IMG008 (*ura1Δ*::*SjURA9*^C265S^). In addition, a point mutation in the corresponding serine codon of *KmURA9* was introduced to change it to a cysteine, yielding strain IMG005 (*ura1Δ*::*KmURA9*^S263C^).

**Fig. 3:**
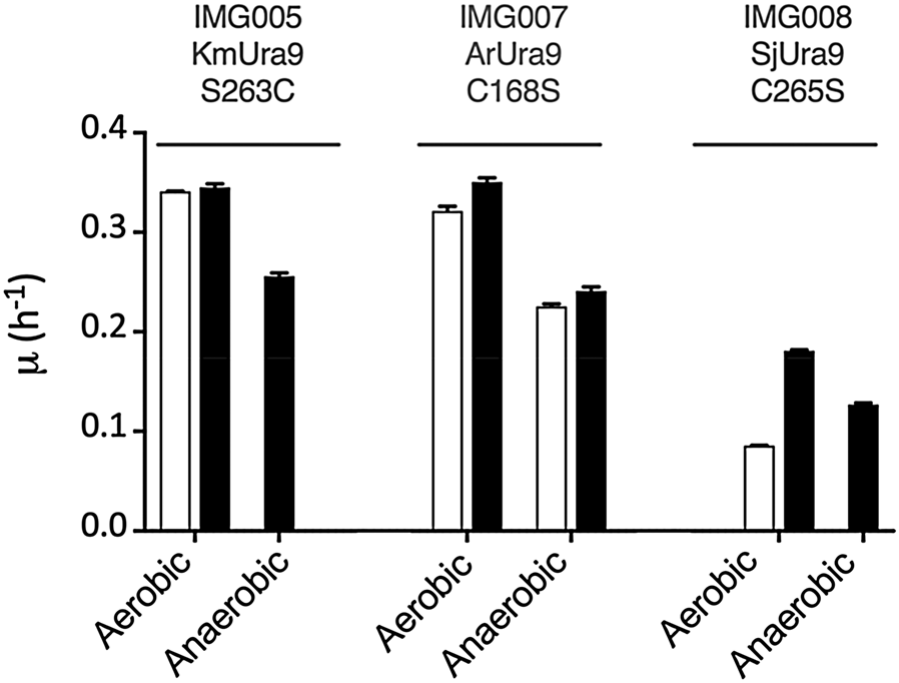
Complementation of uracil auxotrophy of *S. cerevisiae ura1Δ* strains by expressing mutated heterologous *URA9* orthologs. The native *ScURA1* gene of *S. cerevisiae* was replaced by *URA9* orthologs from *K. marxianus* (Km), *A. robustus* (Ar) or *Sch. japonicus* (Sj) with single-nucleotide mutations affecting a specific serine or cysteine residue. Open bars indicate specific growth rates (μ) on synthetic medium without uracil (SMUD), closed bars indicate μ in uracil-supplemented synthetic medium (SMUD + ura). Cultures were grown aerobically or anaerobically as indicated in the Figure. Relevant genotypes of *S. cerevisiae* strains: IMG005; *ura1Δ*::*KmURA9*^S263C^, IMG007; *ura1Δ*::*Arura9*^C168S^, IMG008; *ura1Δ*::*SjURA9*^C265S^. Data represent the average from biological duplicates and mean deviation (Additional file 5: Table S1)

Changing the active-site serine residue (S263) of KmUra9 to a cysteine did not affect aerobic growth of *S. cerevisiae*, as shown by identical specific growth rates of strains IMG005 (*ura1Δ*::*KmURA9*^S263C^) and IMI446 (*ura1Δ*::*KmURA9*) on SMUD (Fig. 3, Additional file 5: Table S1). This result indicated that, at least in KmUra9, the serine catalytic base that is strongly conserved in canonical Class-II DHODs is not essential for activity under aerobic conditions. However, strain IMG005 did not grow anaerobically without uracil supplementation (Fig. 3, Additional file 5: Table S1), indicating that replacement of the catalytic-base serine residue of KmUra9 by a cysteine is not sufficient to enable anaerobic functionality in *S. cerevisiae*.

Replacing the active-site cysteine residue in ArUra9 by a serine residue did not cause a different aerobic or anaerobic growth rate on SMUD of strain IMG007 (*ura1Δ*::*Arura9*^C168S^) relative to its parental strain (Fig. 3, Additional file 5: Table S1). Apparently, despite our observation that an active-site cysteine residue occurred in different anaerobically active Ura9 orthologs, it is not required for anaerobic functionality of ArUra9 in *S. cerevisiae*. A strikingly different result was obtained upon changing the cysteine residue in the active site of SjUra9 to a serine. Under aerobic conditions, exponential growth of strain IMG008 (*ura1Δ*::*SjURA9*^C265S^) on SMUD was nearly two-fold slower than that of its parental strain IMI452 (*ura1Δ*::*SjURA9*). In contrast, strain IMG008 (*ura1Δ*::*SjURA9*^C265S^) failed to grow on SMUD under anaerobic conditions. These results indicated that the active-site cysteine residue in SjUra9, but not in ArUra9, is required for DHOD activity under anaerobic conditions.

### Subcellular localization of heterologous Ura9 orthologs expressed in *S. cerevisiae*

**Fig. 4:**
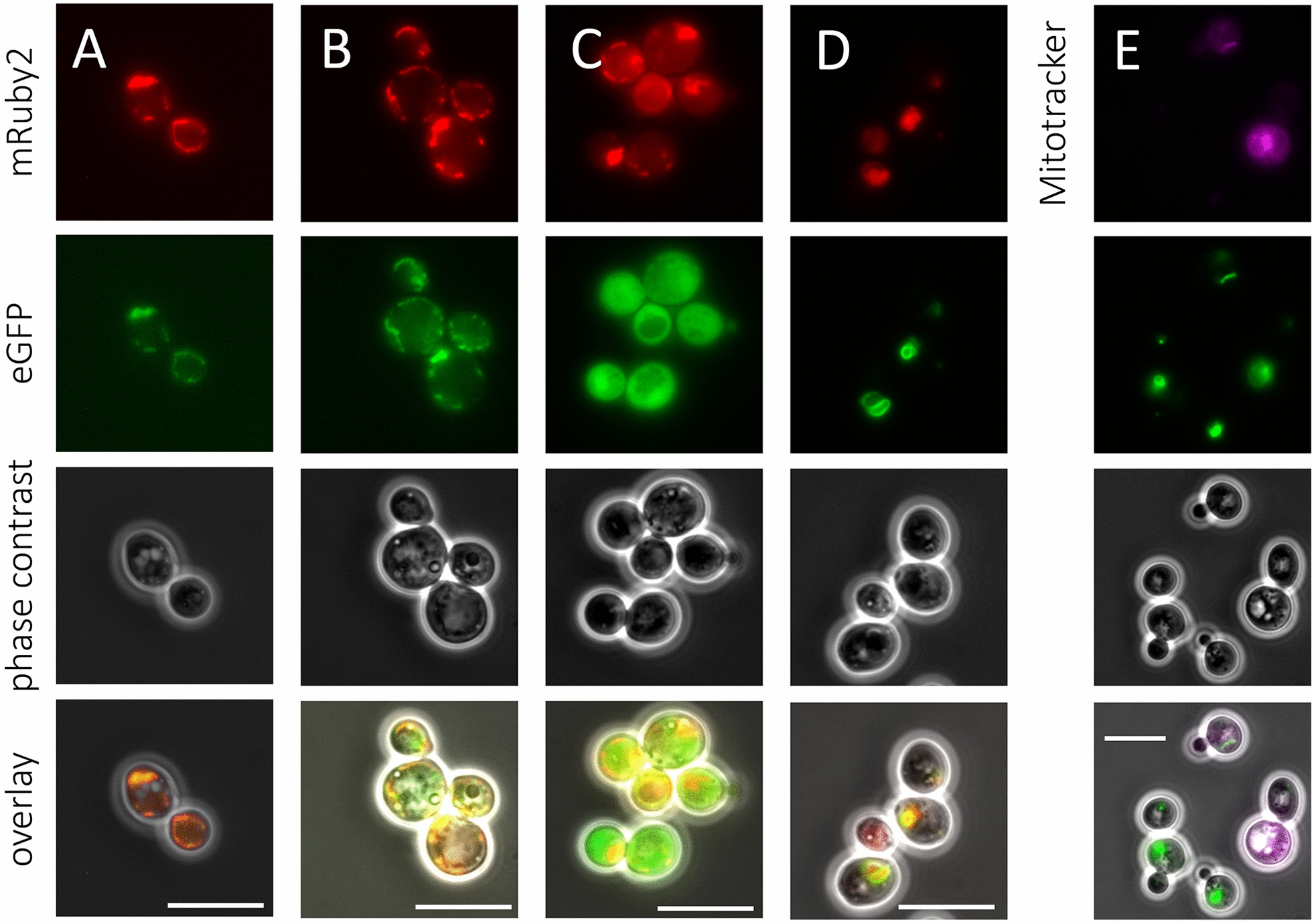
Fluorescence microscopy images of *S. cerevisiae* strains expressing mitochondrially targeted mRuby2 together with different Ura9 orthologs fused to eGFP. Cells were grown on SMUD for at least two duplications and fluorescence of eGFP, mRuby and MitoTracker Deep Rep FM was detected by fluorescence microscopy. For each strain, from top to bottom: mRuby2/MitoTracker Deep Red FM fluorescence specifically localized to mitochondria, indicating localization of mitochondrial mass; eGFP fluorescence, tagged to different Ura9 orthologs, indicating subcellular Ura9 localization; phase-contrast image; and an overlay of all channels. From left to right; **A** IME604, expressing *OpURA9-eGFP*, **B** IME601 (*DbURA9-eGFP*), **C** IME600 (*Arura9-eGFP*), **D** IME602, (*SjURA9-eGFP*) and **E** IME602 stained with MitoTracker Deep Red FM. Scale bars are equivalent to 10 μm. Pictures are a representation of the full culture

To investigate subcellular localization of Ura9 orthologs, eGFP fusions of anaerobically active ArUra9, DbUra9 and SjUra9, as well as of OpUra9, were expressed from multicopy (mc) plasmids in an *S. cerevisiae ura1Δ* strain, followed by fluorescence-microscopy analysis of the resulting strains (Fig. 4). A co-expressed mRuby2 fluorescent protein fused to the pre*COX4* mitochondrial targeting sequence [49] was used as marker for mitochondrial localization.

In *S. cerevisiae* strains IME600 (*ura1Δ* mc*Arura9-eGFP*), IME601 (*ura1Δ* mc*DbURA9-eGFP*) and IME604 (*ura1Δ* mc*OpURA9-eGFP*), mRuby2 fluorescence showed multiple small mitochondria, a pattern that is representative for respiring cells [50]. Consistent with the localization of canonical eukaryotic Class-II DHODs [5], OpUra9-eGFP fluorescence overlapped with that of pre*COX4*-MTS-mRuby2 (Fig. 4A). A similar co-localization of DbUra9-eGFP and mRuby in strain IME601 indicated that, despite its activity under anaerobic conditions, DbUra9 was targeted to mitochondria (Fig. 4B). Consistent with the N-terminal truncation of Ura9 orthologs from Neocallimastigomycetes, but in striking contrast to canonical eukaryotic Class-II DHODs, ArUra9-eGFP was clearly localized to the yeast cytosol (Fig. 4C).

In strain IME602 (*ura1Δ* mc*SjURA9-eGFP*), mRuby2 fluorescence did not reveal the punctuate mitochondrial structures seen in the other strains. Instead, eGFP fluorescence was associated with tubular structures, that partially overlapped with a less defined mRuby2 fluorescence (Fig. 4D). Although elongated mitochondrial morphologies occur in fermenting *S. cerevisiae* cells [50], the diffuse mRuby2 fluorescence in strain IME602 did not allow for clear localization of SjUra9-eGFP. This strain was therefore also stained with the dye MitoTracker Deep Red. This approach only yielded vague tube-like structures or no fluorescence at all (Fig. 4E). Since staining by both pre*COX4*-MTS-mRuby2 and MitoTracker Deep Red depend on mitochondrial membrane potential [49], we hypothesized that expression of *SjURA9* reduces or abolishes mitochondrial membrane potential, possibly as a consequence of a loss of respiratory capacity [51].

### Expression of *SjURA9* in *S. cerevisiae* causes loss of respiratory capacity and mitochondrial DNA

**Fig. 5:**
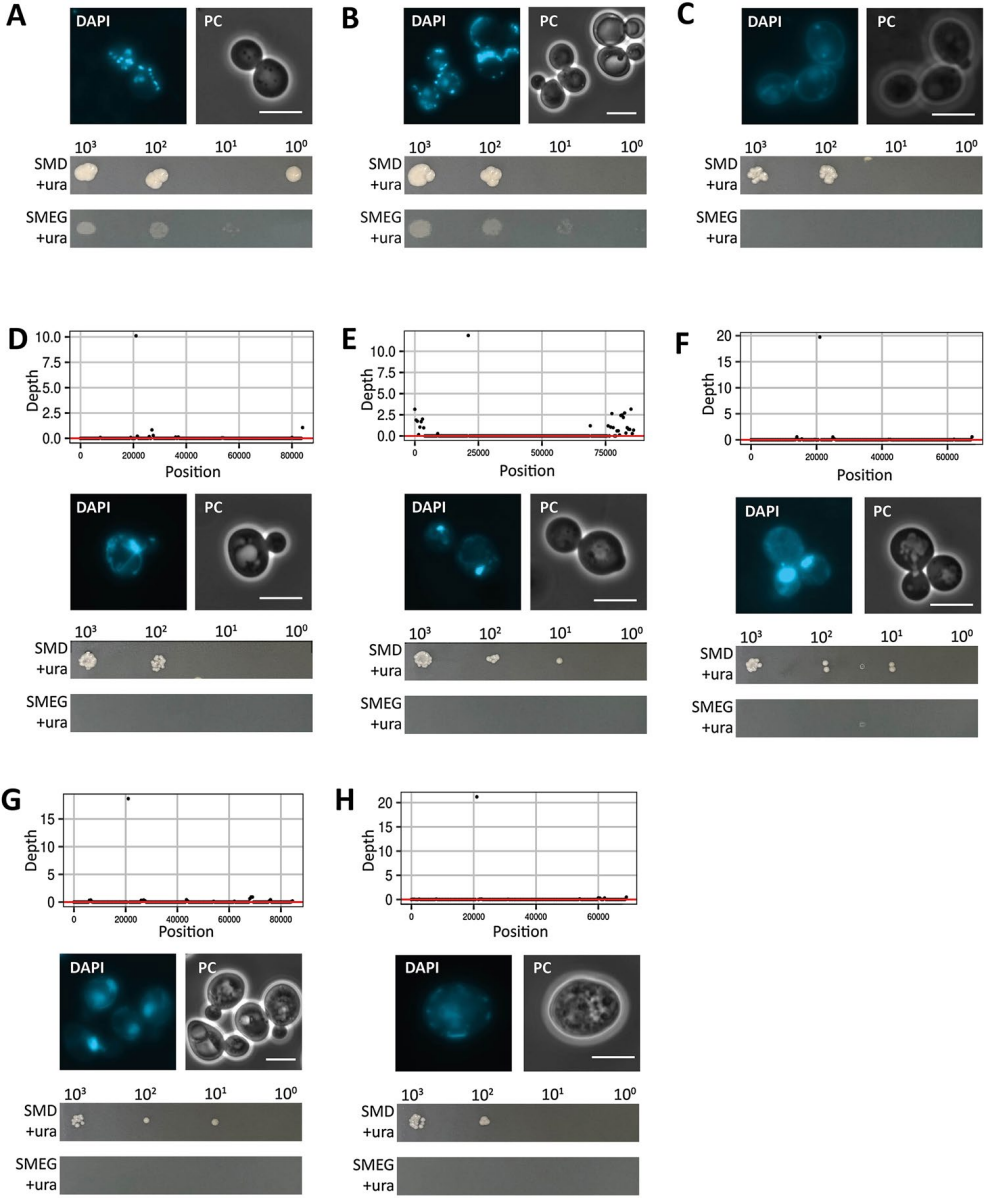
Mitochondrial DNA integrity and respiratory capacity of strains expressing *SjURA9*. Top: sequencing coverage depth (average of non-overlapping 500 bp sliding window of the mitochondrial genome) obtained by short-read sequencing of mitochondrial DNA relative to the reference strain CEN.PK113-7D. Middle: phase contrast (PC) microscopy and fluorescence of nuclei and mitochondrial nucleoids of yeast cells stained with the DNA-specific fluorescent dye DAPI; scale bars represent 5 μm. Bottom: spot plate assays on glucose-containing synthetic medium with uracil (SMUD + ura) and ethanol-glycerol containing synthetic medium with uracil (SMEG + ura). SMUD + ura and SMEG + ura plates were incubated at 30 °C for 3 and 10 days respectively. Panels show data for the following yeast strains: **A** *S. cerevisiae* CENPK.113-7D (reference), **B** *S. cerevisiae* IME603 (multicopy *ScURA1*), **C** *S. cerevisiae* IMK242 (*rip1Δ::kanMX*), **D** *S. cerevisiae* IMI452 (*ura1Δ*::*SjURA9,* from IMX585), **E** *S. cerevisiae* IMI462 (*ura1Δ*::*SjURA9,* from IMX2600), **F** *S. cerevisiae* IMG008 (*ura1Δ*::*SjURA9*^C265S^), **G** *S. cerevisiae* IME571 (multicopy *SjURA9*) and, **H** *S. cerevisiae* IMS1206 (IME571 cured from its *SjURA9* expression plasmid)

To investigate whether expression of *SjURA9* causes loss of respiratory capacity, *SjURA9*-expressing *S. cerevisiae* strains were tested for their ability to grow on non-fermentable carbon sources. In contrast to the reference strain *S. cerevisiae* CEN.PK113-7D (Fig. 5A), three independently constructed strains expressing *SjURA9* (IMI452, IMI462 and IME571; Additional file 5: Table S2) failed to grow on synthetic medium supplemented with ethanol and glycerol (SMEG, Fig. 5D, E, F, G, respectively). The inability of *SjURA9*-expressing strains to grow on SMEG resembled that of the respiratory-deficient strain IMK242 [52]. In contrast, strain IME603, which expressed *ScURA1* from a multicopy plasmid, as well as *ura1Δ* strains expressing other *URA9-*expressing did grow on SMEG, (Additional file 5: Figure S3). Removal of the *SjURA9* expression plasmid from strain IME571, yielding strain IMS1206, did not restore growth on these non-fermentable carbon sources (Fig. 5H).

To explore whether the loss of respiratory competence in *SjURA9*-expressing strains was caused by loss of mitochondrial DNA, its presence was evaluated by staining with the DNA-specific dye 4′,6-diamidino-2-phenylindole (DAPI, [53]) and by whole-genome sequencing. The reference *S. cerevisiae* strain CEN.PK113-7D (Fig. 5A) and a strain expressing *ScURA1* from a multicopy plasmid (IME603; Fig. 5B) showed punctuated extranuclear DAPI staining. The *SjURA9-*expressing *S. cerevisiae* strains IMI462, IMG008 and IME571 only showed fluorescent staining of nuclear DNA, while strain IMI452 (*ura1Δ::SjURA9*) showed vague DAPI-stained tubular structures. Whole-genome sequencing showed absence of mitochondrial DNA in all the four strains *SjURA9*-expressing *S. cerevisiae* strains (Fig. 5). These observations indicated that expression of *SjURA9* in aerobic *S. cerevisiae* cultures causes loss of mitochondrial DNA and respiratory capacity.

### ArUra9 uses free FAD and FMN as electron acceptors

The absence of mitochondria in Neocallimastigomycetes and the cytosolic localization of ArUra9-eGFP in *S. cerevisiae* implied that its activity was unlikely to involve components of the mitochondrial respiratory chain. To identify possible natural electron acceptors of ArUra9, enzyme-activity assays were performed in cell extracts of strain IME569 (*ura1Δ* mc*Arura9*). Cell extracts of strains IME603 (mc*ScURA1*) and IMK824 (*ura1Δ*) were included as references.

Consistent with literature reports [54], cell extracts that only contained the Class-I DHOD ScUra1 (strain IME603) showed dihydroorotate oxidation with fumarate as electron acceptor (0.11 μmol∙mg protein^−1^.min^−1^), while a similar activity was observed with the artificial electron acceptor phenazine methosulfate (PMS, Table 3). ScUra1-containing cell extracts also showed dihydroorotate oxidation without addition of electron acceptor (Table 3), which was attributed to the previously reported ability of DHODs to use molecular oxygen as electron acceptor [55–58].

**Table 3:** Dihydroorotate-dehydrogenase activities in cell extracts of *S. cerevisiae* strains expressing different DHODs, measured with different electron acceptors

Electron acceptor	DHOD activity (μmol·mg protein^−1^·min^−1^)		
	*S. cerevisiae* IMK824 *ura1Δ*	*S. cerevisiae* IME603 mc*ScURA1*	*S. cerevisiae* IME569 *ura1Δ* mc*Arura9*
–	< 0.005	0.010 ± 0.001	0.008 ± 0.001
PMS	< 0.005	0.111 ± 0.007	0.132 ± 0.029
Fumarate	< 0.005	0.110 ± 0.020	0.006 ± 0.000
NAD^+^	< 0.005	< 0.005	< 0.005
NADP^+^	< 0.005	< 0.005	< 0.005
Q_D_	< 0.005	0.033 ± 0.022	0.009 ± 0.003
FMN	< 0.005	0.037 ± 0.002	0.126 ± 0.012
FAD	< 0.005	0.011 ± 0.002	0.148 ± 0.032

Activities were measured in *S. cerevisiae* strains IMK824 (*ura1Δ*), IME603 (expressing *ScURA1* from a multicopy plasmid) and in the strain IME569 (expressing *Arura9* from a multicopy plasmid). Activities were measured without addition of electron acceptor (-), 0.1 mM phenazine methosulfate (PMS), 1 mM fumarate, 1 mM nicotinamine adenine dinucleotide (NAD^+^), 1 mM nicotinamide adenine dinucleotide phosphate (NADP^+^), 0.1 mM decylubiquinone (Q_D_), 20 μM flavin mononucleotide (FMN) or 20 μM flavin adenine dinucleotide (FAD). Activities are represented as the average ± mean deviation of activities measured with two independently prepared cell extracts

A high rate of PMS-dependent dihydroorotate oxidation (0.13 μmol∙mg protein^−1^∙min^−1^) confirmed DHODase activity in cell extracts of the ArUra9-expressing strain IME569. A low activity in the absence of an added electron acceptor (Table 3) suggested that, like other DHODs, ArUra9 can use molecular oxygen as electron acceptor. Assays in which fumarate, NAD^+^, NADP^+^ or decylubiquinone were added to reaction mixtures did not increase activities beyond this basal level. Other compounds with a standard redox potential above or close to that of DHOD-bound FMN cofactors (− 242 to − 310 mV; [58–60]) were therefore tested. Addition of flavin adenine mononucleotide (FAD; E’^0^ = − 219 mV [61]), flavin mononucleotide (FMN; E’^0^ = − 219 mV [61]) strongly promoted dihydroorotate oxidation by cell extracts of the ArUra9-expressing strain IME569, with DHOD activities of 0.15 μmol∙mg protein^−1^·min^−1^ and 0.13 μmol∙mg protein^−1^·min^−1^, respectively. Supplementation of dihydroxyacetone phosphate (DHAP), acetaldehyde, pyruvate or oxaloacetate as electron acceptor did not increase enzyme activity (Additional file 5: Table S4).

## Discussion

Fumarate-dependent Class-IA DHODs in *S. cerevisiae* (ScUra1) and closely related yeasts circumvent the oxygen requirement of respiration-dependent pyrimidine synthesis via the mitochondrial Class-II DHODs that occur in most other fungi [19, 20, 22]. The assumption that presence of a Class-IA DHOD is required for anaerobic pyrimidine prototrophy in fungi was first called into question when analysis of the genome of *D. bruxellensis* indicated absence of a Class-IA DHOD gene and, instead, revealed a sequence (*DbURA9*) with similarity to Class-II DHOD genes [17, 23, 28]. We identified similar situations in obligately anaerobic Neocallimastigomycetes and in the facultatively anaerobic fission yeast *Sch. japonicus* (Table 1, Fig. 1). Heterologous expression studies in *ura1Δ S. cerevisiae* showed that DbUra9, as well as orthologs from the Neocallimastigomycete *A. robustus* (ArUra9) and *Sch. japonicus* (SjUra9), supported anaerobic pyrimidine prototrophy. This phenotype was not observed for *ura1Δ S. cerevisiae* strains expressing *URA9* genes from the aerobic yeasts *K. marxianus* and *O. parapolymorpha* (Fig. 2) nor in similar experiments involving *URA9* genes from other aerobic yeasts [8, 19, 25].

A cysteine as catalytic base is considered a hallmark of Class-I DHODs [57] while, with few reported exceptions, Class-II enzymes have a serine in this position [16]. Instead, the three anaerobically active Ura9 orthologs investigated in this study had a Cys in the catalytic-base position and shared this feature with only 3 of 331 other predicted fungal Ura9 orthologs. The latter three sequences originated from *C. reversa*, *Sm. culicis* and *G. prolifera* (Fig. 1), which were all originally isolated from microaerobic or anoxic environments (dung [62], insect guts [63] and submerged fruits [64], respectively). A Cys-to-Ser change in the active site of *Sch. japonicus* SjUra9 specifically abolished its ability to support anaerobic pyrimidine prototrophy of *S. cerevisiae* (Fig. 3). While this result suggested that a Cys as catalytic base can be relevant for anaerobic functionality, changes at the same position of *A. robustus* ArUra9 (Cys to Ser) and *K. marxianus* KmUra9 (Ser to Cys) showed it is neither absolutely required nor sufficient for anaerobic activity of Class-II DHODs (Fig. 3).

Enzyme assays in cell extracts showed that ArAro9 can use free FAD and FMN as electron acceptors (Table 3), which was not previously observed for DHODs. Biochemical standard redox potentials of the non-enzyme-bound FADH_2_/FAD and FMNH_2_/FMN redox couples (E^0^’ = − 219 mV; [65]) and those of DHOD-bound FMNH_2_/FMN (− 242 to − 330 mV; [58–60]) are compatible with either of these flavin cofactors acting as physiological electron acceptor of ArUra9. In anaerobic chemostat cultures of *S. cerevisiae*, intracellular FAD and FMN contents of 0.17 and 0.09 μmol (g biomass)^−1^ were reported [66]. While use of free flavins in cellular redox reactions is relatively rare, the *S. cerevisiae* fumarate-reductase Osm1 can re-oxidize free FADH_2_ [67]. Since combined deletion of *OSM1* and its paralog *FRD1* abolishes anaerobic growth of *S. cerevisiae* [68], we could not experimentally verify their involvement in the in vivo anaerobic activity of ArUra9. Sequences with strong homology to *S. cerevisiae* Frd1 and/or Osm1 were found in Neocallimastigomycetes, *Sch japonicus* and *D. bruxellensis* (Additional file 5: Table S3). In silico prediction of subcellular localization indicated that these putative fumarate reductases were mitochondrial in *Sch. japonicus* and *D. bruxellensis*. Consistent with the inferred cytosolic localization of ArUra9, they were predicted to occur in the cytosol of Neocallimastigomycetes (Additional file 5: Table S3).

Previous studies implicated the N-terminal domains of Class II DHODs in quinone binding [46, 69]. Based on analysis of 1500 Class II DHOD sequences and structural alignments, Sousa et al*.* [16] proposed conserved residues involved in quinone binding, stabilizing and pocket entry (Additional file 5: Figure S2). Ura9 sequences from Neocallimastigomycetes lacked several of these conserved residues as a consequence of their N-terminal truncation. In addition, a conserved positively-charged residue implicated in quinone binding by other Class-II DHODs was replaced by a hydrophobic residue in ArUra9 and orthologs from other Neocallimastigomycetes (Additional file 5: Figure S2). These observations, together with the absence of quinone-dependent DHOD activity in cell extracts containing ArUra9 (Table 3) support the notion that, even though quinones have been detected in these anaerobes [70], they are not involved in the activity of ArUra9 and orthologs in other Neocallimastigomycetes,.

Consistent with the N-terminal truncation of Ura9 orthologs from Neocallimastigomycetes (Additional file 5: Figure S2), an ArUra9-eGFP fusion expressed in *S. cerevisiae* was localized to the cytosol (Fig. 4). *Sch. japonicus* and *D. bruxellensis* Ura9 orthologs retained an N-terminal mitochondrial targeting sequence, as well as conserved residues proposed to be involved quinone binding (Additional file 5: Figure S2). The only difference in quinone-associated residues was a Tyr-to-Phe change in SjUra9 (Y137), which, in view of the similarity of these amino acids, may not have affected functionality (Additional file 5: Figure S2). We were unable to measure activities of DbUra9 and ScUra9 in cell extracts with the artificial electron acceptor PMS or other potential electron acceptors.

DbUra9-expressing *ura1Δ* isolates of *S. cerevisiae* evolved for fast anaerobic pyrimidine-prototrophy revealed three different mutations in the *FUM1* fumarase gene. Mutations in the human fumarase gene (*FH*) have been implicated with different types of cancer due to increased fumarate concentrations [71]. Thr218 and Ala294 of DbUra9 correspond to Val197 and Ala274, respectively, in FH and are both located in highly conserved regions [72, 73], while Ala274 resides in the active site [74]. Mutation of Ala274 to a valine in human fumarate hydratase (FH) was implicated in ovarian mucinous cystadenoma [75], and resulted in a 50% decreased activity of the enzyme [73]. By analogy, it seems probable that the Fum1^A294V^ change in strain IMS1169 also led to a reduced fumarase activity. Corresponding amino acids in human FH of Thr218 and Met432 are located in the core helix, and accumulated fumarate resulting from mutations in this helix have been associated with different types of cancer [74]. If further research confirms higher intracellular fumarate concentrations in strains carrying *FUM1* mutations, these might favor in vivo use of fumarate as electron acceptor, as in Class I-A enzymes [57] or, alternatively, stimulate reoxidation of FADH_2_ via fumarate reductases.

Anaerobic pyrimidine synthesis, combined with acquisition by horizontal gene transfer of genes enabling sterol-independent anaerobic growth (squalene hopene cyclase; [31]) and anaerobic deoxynucleotide synthesis (Class-I ribonucleotide reductase; [41]), indicates that *Sch. japonicus* is remarkably well adapted to anaerobic growth. The observation that independently constructed SjUra9-expressing *S. cerevisiae* strains consistently showed loss of respiratory capacity and mitochondrial DNA (Fig. 5) indicated a trade-off between anaerobic pyrimidine synthesis and respiratory competence. Quinone-dependent DHODs are known to react with oxygen, leading to formation of hydrogen peroxide and superoxide [55]. In *S. cerevisiae*, these reactive oxygen species have been implicated in loss of respiratory capacity [76]. Despite presence of respiratory proteins, including low levels of all cytochromes [76], *Sch. japonicus* strains are naturally respiratory deficient [77–79]. It is tempting to speculate whether this phenotype may have provided a driving force for evolution of a respiration-independent DHOD or, alternatively, be a consequence of evolution of SjUra9 for anaerobic functionality. With respect to the latter option, it should be considered that the heterologous context in which loss of respiratory competence was observed in this study does not necessarily reflect conditions in *Sch. japonicus* or its ancestors.

## Conclusions

Our results show that, in addition to the well-established acquisition of a Class-I DHOD by *S. cerevisiae* and closely related yeasts, at least three separate events in fungal evolution enabled anaerobic pyrimidine synthesis by variants of Class-II DHODs that do not depend on aerobic respiration. These anaerobically active variants were shown to have a cysteine instead of a conserved serine residue in their catalytic sites. Their in vivo activities were not dependent on aerobic respiration, and, in Neocallimastigomycetes, they were not membrane associated and could use free FAD or FMN as electron acceptor. These remarkable differences with canonical Class-II DHODs underline the plasticity of fungal genomes and genes under selective pressure and extend our knowledge on eukaryotic adaptation to anoxic environments.

## Methods

### Yeast strains, media and strain maintenance

*Saccharomyces cerevisiae* strains were derived from the CEN.PK lineage (Additional file 5: Table S2; [80, 81]). *O. parapolymorpha* CBS11895, *K. marxianus* CBS6556 and *Sch. japonicus* CBS5679 were obtained from the Westerdijk Institute (Utrecht, The Netherlands). Synthetic media with ammonium as nitrogen source (SM) and with urea as nitrogen source (SMU), containing vitamins and trace elements, were prepared and sterilized as described previously [82, 83]. A separately autoclaved (30 min, 110 °C) d-glucose solution (50% w/v) was added to sterile SMU or SM at a concentration of 20 g L^−1^, yielding SMUD and SMD, respectively. SM with ethanol and glycerol (SMEG) and complex yeast extract-peptone-glucose medium (YPD) were prepared as described previously [84]. Where indicated, YPD was supplemented with 200 mg L^−1^ filter-sterilized geneticin (G418; Thermo Fisher Scientific, Waltham, MA) or hygromycin B (HygB; Thermo Fisher Scientific). Synthetic media for anaerobic growth experiments were supplemented with Tween 80 and ergosterol [82]. Uracil-auxotrophic strains were routinely grown on SMUD supplemented with 150 mg L^−1^ uracil (SMD + ura) or, to obtain uracil-limited pre-cultures, with 15 mg L^−1^ uracil (SMUD + ura0.1). Solid synthetic and complex media were prepared by adding 20 g L^−1^ Bacto agar (Difco laboratories Inc, Detroit, MI) prior to autoclaving. *Escherichia coli* strains were grown on Lysogeny Broth (LB; [85]), supplemented with 100 mg L^−1^ filter-sterilized ampicillin (Sigma Aldrich, St. Louis, MO) or chloramphenicol (Sigma Aldrich) as indicated (LB-amp and LB-cam, respectively). Frozen stock cultures of yeast strains were prepared as described previously [86] after growth to mid-exponential phase at 30 °C on YPD (strains CENPK.113-5D, CEN.PK113-7D, IMX585, IMX2600, CBS6556 and CBS11895), on SMUD + ura (strain IMK824) or on SMUD (other strains). *E. coli* strains were grown at 37 °C on LB-amp or LB-cam and frozen stock cultures were prepared as described by Mans et al*.* [86].

### Molecular biology techniques

Phusion High Fidelity DNA Polymerase (Thermo Fisher Scientific) and PAGE-purified oligonucleotide primers (Additional file 5: Table S5, Sigma Aldrich) were used in polymerase chain reactions (PCRs) for cloning and sequencing. Diagnostic PCRs were performed with desalted oligonucleotides (Additional file 5: Table S5, Sigma Aldrich) and DreamTag Mastermix 2X (Thermo Fisher Scientific). Genomic DNA as template for PCRs was isolated using a YeaStar Genomic DNA kit (Zymo Research, Irvine, CA). PCR products were purified with a GeneElute PCR Clean-Up kit (Sigma Aldrich) or from 1% agarose gels using a Zymoclean Gel DNA Recovery Kit (Zymo Research). Gibson Assembly of purified DNA fragments with 20 bp sequence overlaps was performed with the NEBuilder HiFi DNA Assembly Mastermix (New England Biolabs, Ipswich, MA). Golden-Gate assembly was performed according to Lee et al*.* [87]. *E. coli* XL1-Blue (Agilent Technologies, Santa Clara, CA) was chemically transformed following manufacturer’s instructions and plated on selective media. Correct plasmid assembly was verified by diagnostic PCRs on *E. coli* transformants [86]. Cas9-mediated genome editing in *S. cerevisiae* was performed according to Mans et al*.* [86]. The LiAc/SS-DNA/PEG method [88] was used for yeast transformation with plasmids (at least 1 μg DNA per transformation) or linear DNA fragments (0.5–1 μg per transformation). Single-colony isolates of randomly picked transformants were obtained by three re-streaks on selective media. Integrations and deletions were verified by diagnostic PCRs with genomic DNA as a template. Construction of plasmids and yeast strains is described in detail in Additional file 6.

### Whole-genome DNA sequencing

Genomic DNA of yeast strains was isolated from overnight cultures on YPD, except for strain IME571 that was grown on SMUD, using the Qiagen Genomic DNA 100/G kit (Qiagen, Hilden, Germany) with the Proteinase-K step extended to 3 h. DNA concentrations were measured on a Qubit Fluorometer (Invitrogen, Carlsbad, CA, USA) with the QuBit BR dsDNA Assay kit (Invitrogen). Whole-genome sequencing on an Illumina MiSeq platform (Illumina Novoseq 6000, Illumina Inc., San Diego, CA, USA) was performed by Novogene Europe (Cambridge, UK; strains IMI439, IMS1167, IMS1168, IMS1169 and IMS1170), Macrogen Europe BV (Amsterdam, The Netherlands; strains IMG008, IME571 and IMS1206) or in-house (strains IMI452 and IMI462). For in-house sequencing, the Nextera DNA Flex 509 Library Prep kit (Illumina) was used for paired-end library preparation. Genome sequences were deposited at GenBank (BioProject accession number PRJNA745202).

### Whole-genome sequence analysis

Sequencing data were processed as described previously [89]. For sequence analysis of the evolved strains IMS1167-IMS1170, their parental strain IMI439 was used as reference to obtain sequence differences. For IMI452/IMI462/IMG008 or IME571/IMS1206 IMI452 and IME571 were used as reference, respectively. Identified SNP’s were individually checked with the Integrated Genomics Viewer (IGV; [90]). Mitochondrial DNA coverage plots were generated by calculating the average of non-overlapping 500 bp sliding windows (R version 3.6.0).

### Protein sequence homology search and phylogenetic analysis

Proteomes of *A. robustus* (NCBI taxid 1754,192), *P. finnis* (taxid 1754191), *Neocallimastix californiae* (1754190), *Sch. japonicus* (402676), *D. bruxellensis* (5007; [91]), *K. marxianus* (1003335) and *O. parapolymorpha* (871575) were subjected to protein blast search in NCBI [92], using LkUra1 (UniProt KB accession number Q7Z892) and LkUra9 (Q6V3W9) as queries and applying default settings. Similarly, the proteomes of *A. robustus*, *P. finnis*, *N. californiae*, *Sch. japonicus* and that of *D. bruxellensis* were subjected to a protein blast using ScFrd1 (GenBank accession number EIW10990.1) or Osm1 (EIW09573.1) as query, default settings were applied.

To predict the localization of putative fumarate reductases in *A. robustus*, *N. californiae*, *P. finnis, D. bruxellensis* and *Sch. japonicus*, protein sequences were compared to known fungal sorting signals and motifs using the online computational tool WoLF PSORT [93]. The highest scoring cellular component for fungal settings was retrieved.

Bacterial and fungal amino acid sequences available from UniProt reference proteomes release 2019_02 [94] were systematically searched for Class-II dihydroorotate dehydrogenase orthologs. The database of fungal reference proteomes (NCBI taxid 4751) was supplemented with sequences available from the UniProt trembl division for the following organisms: *D. bruxellensis* (5007), *Komagataella phaffii* (981350), *Komagataella pseudopastoris* (169507), *Komagataella pastoris* (4922), *Ogataea polymorpha* (460523), *Pichia membranifaciens* (763406), *Pichia kudriavzevii* (4909), *Neocallimastix californiae* (1754190)*, P. finnis* (1754191)*, A._robustus* (1754192) and *Piromyces* sp. E2 (73868). Then, a Class-II DHOD of *L. kluyveri* CBS3082 (LkUra9; UniProt KB accession number Q6V3W9)[19] was used as query for a HMMER search [39], using cutoff values of 1e-5 and requiring hits to correspond to at least 75% of the query sequence length resulting in 724 fungal and 1595 bacterial Ura9 homologs. The sets of Ura9 homologs were further used to select a set of orthologs. For this purpose, the database of fungal proteomes was used to calculate all possible co-ortholog sets with proteinortho v6.0.25 [95] running diamond v2.0.8 [96], obtaining 331 Ura9 orthologs (Additional file 1). Similarly, the search for bacterial Ura9 orthologs resulted in 73 sequences (Additional file 1). Ura9-orthologous amino-acid sequences were then subjected to multiple sequence alignment using MAFFT v7.40286 [97] in “einsi” mode. Alignments were trimmed using trimAl v1.287 [98] in “gappyout” mode, and used to build a phylogenetic tree with RAxML-NG v0.8.188 [99] using 10 random and 10 parsimony starting trees, 100 Felsenstein Bootstrap replicates, and LG model. The resulting phylogenetic tree was visualized using iTOL (Interactive Tree Of Life) tool v6 [100].

Multiple sequence alignment of selected Ura9-orthologs was performed in Clustal Omega [101] with default settings. Protein sequences of Ura9 orthologs were retrieved from the Uniprot database for *Sch. pombe* (SpUra3; Uniprot KB accession number P32747); *L. kluyveri* (LkUra9; Q6V3W9), *O. parapolymorpha* (OpUra9; W1QJ07), *K. marxianus* (KmUra9; Q6SZS6), *E. coli* (EcUra9; P0A7E1), *D. bruxellensis* (DbUra9; I2JUI3), *Sch. japonicus* (SjUra9; B6JXQ5), *A. robustus* (ArUra9; A0A1Y1XN91), *N. californiae* (NcUra9; A0A1Y2ELQ6) and *P. finnis* (PfUra9; A0A1Y1VDI5) and *C. reversa* (CmUra9; A0A2G5BHD4), *Sm. culicis* (ScuUra9; A0A1R1YI62) and *G. prolifera* (GpUra9; A0A139AY32).

### Cultivation of yeast strains

Aerobic shake-flask cultures were grown in an Innova Incubator (New Brunswick Scientific, Edison, NJ) at 30 °C and 200 rpm. Pre-cultures in 100-mL shake flasks with a working volume of 40 mL were inoculated with frozen stock cultures. Primary pre-cultures of yeast strains expressing plasmid-borne DHOD genes were grown on SMUD and those of other yeast strains on SMUD + ura, and were used to inoculate a secondary pre-culture on SMUD (for plasmid expressing strains) or SMUD + ura0.1. Upon reaching late exponential phase, cultures were centrifuged (5 min at 3000 × *g*) and washed twice with demineralized water. Washed cell suspensions were used to inoculate 500-mL shake flasks containing 100 mL of SMUD + ura or SMUD, at an initial optical density at 660 nm (OD_660_) of 0.2.

Anaerobic cultures were grown in 100-mL shake flasks with a working volume of 80 mL. Pre-cultures were grown aerobically on SMUD + ura as described above, until stationary phase, washed twice with sterile demineralized water and transferred to an anaerobic preculture on SMUD + ura0.1, supplemented with Tween 80 and ergosterol. Flasks were incubated on an IKA KS 260 orbital shaker (240 rpm; Dijkstra Verenigde BV, Lelystad, The Netherlands) placed in a Shel Lab Bactron 300 anaerobic workstation (Sheldon Manufacturing Inc, Cornelius, OR) at 30 °C. The gas mixture supplied to the anaerobic workstation contained 10% CO_2_, 5% H_2_ and 85% N_2_. Measures to minimize inadvertent oxygen entry were implemented as described by [102]. When anaerobic precultures reached stationary phase, they were used to inoculate cultures on SMUD and SMUD + ura supplemented with Tween 80 and ergosterol.

For spot-plate experiments, yeast strains were pre-grown on 20 mL SMUD in 100-mL shake flasks, centrifuged (5 min, 3000*g*) and washed twice with demineralized water. Washed cultures were used for cell counts with a Z2 Coulter particle count and size analyzer (Beckman Coulter, Brea, CA) set at particle size 2.5–7.5 μm. Cells were diluted to a final concentration of 2.5∙10^5^ cells mL^−1^, and subsequently diluted to 2.5∙10^4^ cells mL^−1^, 2.5∙10^3^ cells/mL and 2.5∙10^2^ cells mL^−1^. From these four dilutions, 4 μL of each strain and dilution was transferred to SMD, SMD + ura, SMEG and SMEG + ura plates. All strains were pre-grown and plated in duplicate.

### Analytical methods

Extracellular metabolite concentrations were measured by high performance liquid chromatography as described by Verhoeven et al*.* [103] Optical density at 660 nm of aerobic cultures was measured using an Jenway 7200 spectrophotometer (Bibby Scientific, Staffordshire, UK) after accurate dilution to an OD_660_ between 0.1 and 0.3. Anaerobic cultures were first diluted to an optical density at 600 nm (OD_600_) between 0.15 and 0.35, followed by optical densities measurements at 600 nm on an Ultrospec 10 cell density meter (Biochrom, Harvard Biosience, Holliston, MA) that was placed in the anaerobic workstation [102].

### Microscopy analysis and staining

MitoTracker Deep Red FM (Invitrogen) staining was performed on early exponential phase aerobic cultures by adding 250 nM MitoTracker Deep Red FM to a 1-mL culture sample and subsequent incubation in the dark at 37 °C for 15 min. DNA staining was performed on 1-mL samples of early exponential phase, aerobic cultures on 10 mL SMUD in 50-mL vented Greiner tubes (Greiner Bio-One, Kremsmünster, Austria). Cultures were supplemented with 300 nM 4′,6-diamidino-2-phenylindole (DAPI) dihydrochloride (Sigma Aldrich) and incubated in the dark for 10–15 min at 20 °C. Phase-contrast microscopy was performed using a Zeiss Axio Imager Z1 (Carl Zeiss AG, Oberkochen, Germany) that was equipped with a HAL 100 Halogen illuminator, HBO 100 illuminating system and AxioCam HRm Rev3 detector (60 N-C 1’’ 1.0×) (Carl Zeiss AG). The lateral magnification objective 100×/1.3 oil was used with Immersol 518F type F immersion oil (Carls Zeiss AG). Fluoresence of eGFP was detected using filter set 10 (Carl Zeiss AG; excitation bandpass (BP) 470/20, emission 540/25). MitoTracker Deep Red and mRuby2 were imaged with filterset 14 (excitation BP 535/25, emission longpass (LP) 590) and 50 (excitation BP 640/30, emission BP 690/50) respectively (Carls Zeiss AG). For analysis of DAPI dihydrochloride fluorescence, filterset 49 (excitation Short Pass (SP) 380, emission BP 445/50) was used (Carl Zeiss AG). Results were analysed using the Fiji package of ImageJ [104].

### Preparation of cell extracts

Strains carrying multi-copy plasmids expressing dihydroorotate dehydrogenases, were grown to mid-exponential phase in 100-mL shake flask cultures on SMUD. After centrifugation at 3000×*g* and at 0 °C, biomass was resuspended in 4 mL ice-cold 10 mM potassium phosphate buffer (pH 7.5) with 2 mM EDTA and stored at -20 °C. Samples were thawed on ice, centrifuged at 3000×*g* and at 4 °C, washed with 10 mL ice-cold sonication buffer (100 mM potassium phosphate buffer, pH 7.5 with 2 mM MgCl_2_) and resuspended in 4 mL sonication buffer containing 1 tablet of cOmplete Mini protease inhibitor (Sigma Aldrich) per 10 mL buffer. Cell extracts were prepared by sonication and centrifugation as described previously [105]. Bovine serum albumin (Sigma Aldrich) was used as a reference for analyses of protein concentrations in cell extracts [106].

### Dihydroorotate dehydrogenase activity assays in cell extracts

Dihydroorotate dehydrogenase assays were performed at 30 °C in potassium phosphate buffer, (100 mM, pH 7.5) using a Hitachi U-3010 UV/Visible spectrophotometer (Chiyoda, Tokyo, Japan). Formation of orotate or reduction of NAD(P)^+^ was monitored by measuring absorbance at 300 nm (ε = 3.05 mM^−1^ cm^−1^; [14]) or 340 nm (ε = 6.22 mM^−1^ cm^−1^; [107]) respectively, upon addition of 1 mM dihydroorotate to a temperature-equilibrated reaction mixture containing buffer, cell extract and/or either of the electron acceptors fumarate (1 mM), decylubiquinone (Q_D_, 0.1 mM, dissolved in dimethylsulfoxide), nicotinamide adenine dinucleotide (NAD^+^, 1 mM), nicotinamine adenine dinucleotide phosphate (NADP^+^, 1 mM), flavine adenine dinucleotide (FAD, 20 μM), flavin mononucleotide (FMN, 20 μM) or the artificial electron acceptor phenazine methosulfate (PMS, 0.1 mM). Enzyme assays were performed on two separately prepared cell extracts. Reduction potentials of tested electron acceptors mentioned in the text are relative to the standard hydrogen electrode.

## Supplementary Information

**Additional file 1.** Protein sequences Ura9 orthologs.

**Additional file 2.** Protein IDs Ura9 orthologs.

**Additional file 3.** Raw phylogenetic tree.

**Additional file 4.** Codon-optimized protein sequences.

**Additional file 5.** Supplementary tables and figures.

**Additional file 6.** Plasmid and strain construction.

## Data Availability

Figure 1 was made available online (https://itol.embl.de/export/193190253145446711626368835)Whole-genome sequencing data from strains IMS1167, IMS1168, IMS1169, IMS1170, IMI452, IMI462, IMG008, IME571 and IMS1206 was deposited at NCBI (BioProject accession number PRJNA745202).

## Abbreviations

Ar: *Anaeromyces robustus*
Db: *Dekkera bruxellensis*
DHAP: Dihydroxyacetone phosphate
DHOD: Dihydroorotate dehydrogenase
Cr: *Coemansia reversa*
Cys: Cysteine
FAD: Flavin adenine dinucleotide
FH: Fumarate hydratase
FMN: Flavin mononucleotide
GFP: Green fluorescent protein
Gp: *Gonapodya prolifera*
HGT: Horizontal gene transfer
Km: *Kluyveromyces marxianus*
Nc: *Neocallimastix californiae*
Op: *Ogataea parapolymorpha*
Pf: *Piromyces finnis*
PMS: Phenazine methosulfate
Q_D_: Decylubiquinone
Sc: *Saccharomyces cerevisiae*
Scu: *Smittium culicus*
Ser: Serine
SMD: Synthetic dextrose medium
SMEG: Synthetic ethanol/glycerol medium
SMUD: Synthetic urea medium with dextrose
Sj: *Schizosaccharomyces japonicus*
μ: Maximum specific growth rate
