# Correction to: Class‑II dihydroorotate dehydrogenases from three phylogenetically distant fungi support anaerobic pyrimidine biosynthesis

**DOI:** 10.1186/s40694-021-00123-6

**Published:** 2021-11-16

**Authors:** Jonna Bouwknegt, Charlotte C. Koster, Aurin M. Vos, Raúl A. Ortiz-Merino, Mats Wassink, Marijke A. H. Luttik, Marcel van den Broek, Peter L. Hagedoorn, Jack T. Pronk

**Affiliations:** 1 Department of Biotechnology, Delft University of Technology, van der Maasweg 9, 2629 HZ Delft, The Netherlands; 2 Wageningen Plant Research, Wageningen University and Research, Droevendaalsesteeg 1, 6708 PB Wageningen, The Netherlands

## Correction to: Fungal Biol Biotechnol (2021) 8:10 https://doi.org/10.1186/s40694-021-00117-4

Following publication of the original article [1], the authors reported errors in the text of the Results section and in Table 2. It refers to a mutation in a yeast gene as *VPS1*^I410L^ and to the corresponding change in the Vps1 amino-acid sequence as I410L. The correct descriptions should read VPS1^I401L^ and I401L, respectively. This has been corrected with this erratum.
