# Supplementary material for: Class-II dihydroorotate dehydrogenases from three phylogenetically distant fungi support anaerobic pyrimidine biosynthesis
Source: Fungal Biol Biotechnol. 2021 Oct 16;8:10. doi: 10.1186/s40694-021-00117-4 (PMC8520639; doi:10.1186/s40694-021-00117-4)
Supplement: Supplementary file 5 — Additional file 5. Supplementary tables and figures. [file 40694_2021_117_MOESM5_ESM.docx]

# Supporting Material

# Class-II dihydroorotate dehydrogenases from three phylogenetically distant fungi support anaerobic pyrimidine biosynthesis

Jonna Bouwknegt^1^, Charlotte C. Koster^1^, Aurin M. Vos^2^, Raúl A. Ortiz-Merino^1^, Mats Wassink^1^, Marijke A. H. Luttik^1^, Marcel van den Broek^1^, Peter L. Hagedoorn^1^ and Jack T. Pronk^1^*

^1^Department of Biotechnology, Delft University of Technology, van der Maasweg 9, 2629 HZ Delft, the Netherlands

^2^Wageningen Plant Research, Wageningen University and Research, Droevendaalsesteeg 1, 6708 PB Wageningen, the Netherlands

*Corresponding author: Jack T. Pronk, e-mail [j.t.pronk@tudelft.nl](mailto:j.t.pronk@tudelft.nl), tel +31 15 2782416

**This PDF file contains:**

Figures S1-S4

Tables S1-S6

**Additional files:**

Additional file 1: Protein sequences Ura9 orthologs

Additional file 2: Protein IDs Ura9 orthologs

Additional file 3: Raw phylogenetic tree

Additional file 4: Codon-optimized protein sequences

Additional file 6: Plasmid and strain construction

## Supporting Figures

###

Figure S1. Anaerobic growth of *S. cerevisiae* *ura1Δ* strains expressing heterologous *URA9* genes. The native *URA1* gene of *S. cerevisiae* was replaced by *URA9* orthologs of *O. parapolymorpha* (Op) or *D. bruxellensis* (Db). Growth on medium with uracil (SMUD+ura) is indicated by closed circles, and on medium without uracil (SMUD) by open circles. A: *S. cerevisiae* IMI447 (*ura1ΔOpURA9*) and B: *S. cerevisiae* IMI439 (*ura1ΔDbURA9*). Both strains were transferred to fresh SMUD after 76 h. Specific growth rate of strain IMI439 was measured after the transfer (Additional file 4; Table S1). Optical densities are presented as average and mean deviation of data from duplicate cultures.

###
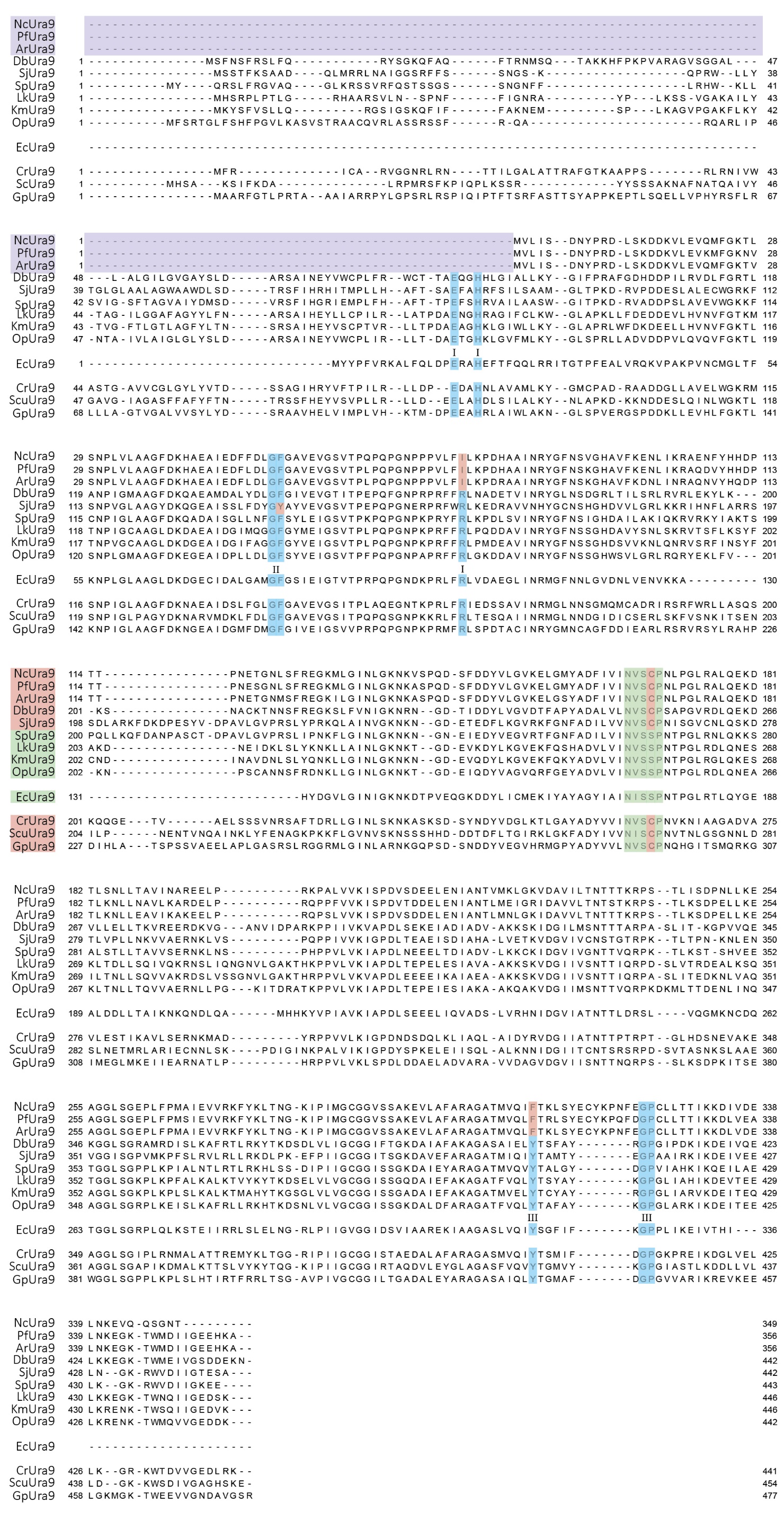

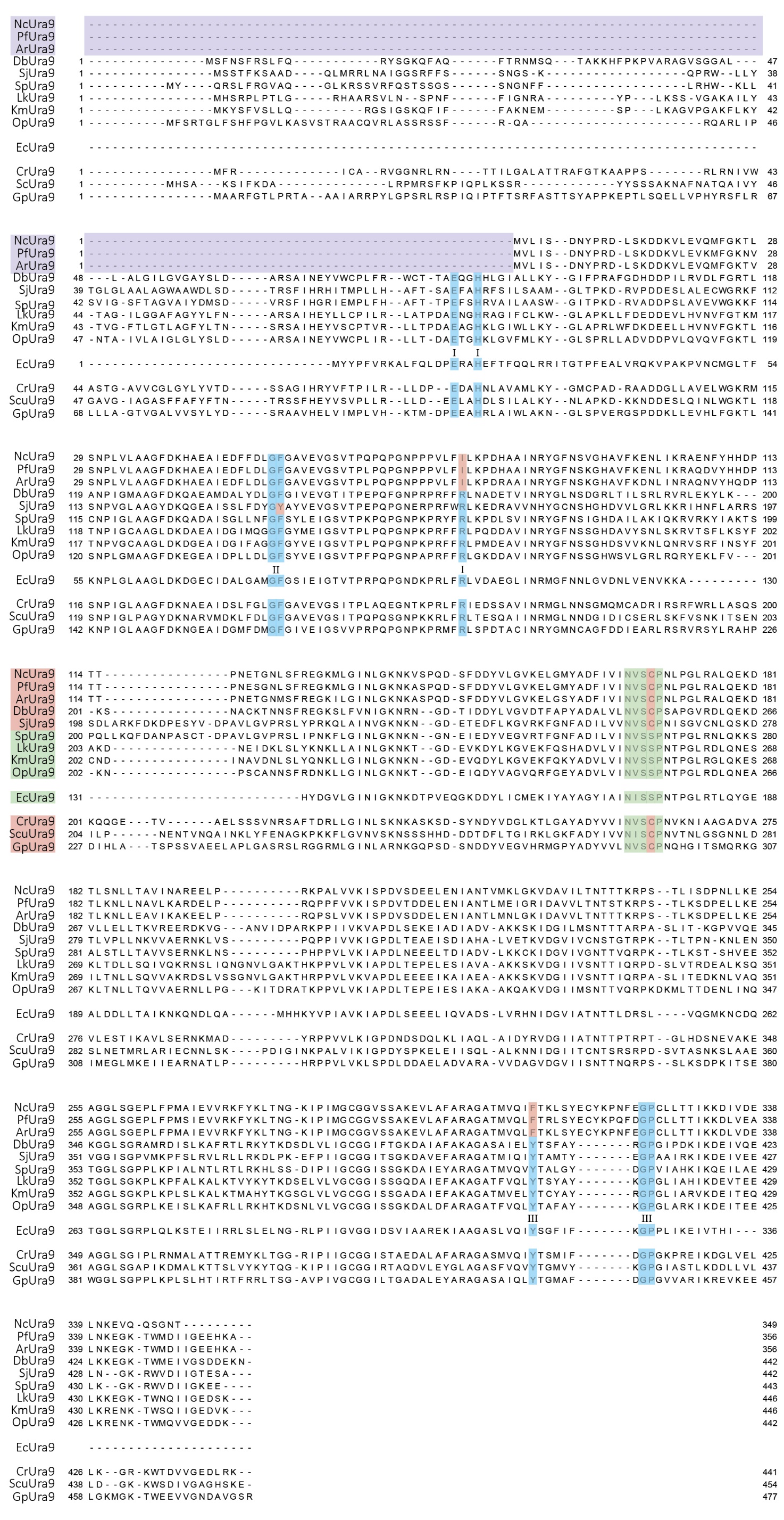
Figure S2. Multiple sequence alignment of Ura9 proteins.

Canonical Class-II DHOD protein sequences of *Sch. pombe* (SpUra3), *L. kluyveri* (LkUra9), *O. parapolymorpha* (OpUra9), *K. marxianus* (KmUra9), *E. coli* (EcUra9), sequences of Ura9 orthologs of the facultatively anaerobic yeasts *D. bruxellensis* (DbUra9) and *Sch. japonicus* (SjUra9), of the Neocallimastigomycetes *A. robustus* (ArUra9), *N. californiae* (NcUra9) and *P. finnis* (PfUra9) and of the fungi *Coemansia reversa* (CmUra9), *Smittium culicis* (ScuUra9) and *Gonopodya prolifera* (GpUra9) were subjected to a multiple sequence alignment in Clustal Omega (default settings). The purple bar indicates an N-terminal truncation of sequences from Neocallimastigomycetes. The conserved active site (NVSSP) is presented in green. Amino acids proposed to be involved in quinone binding (I), stabilizing (II), and entry into the pocket (III) by Sousa *et al.* [1] are shown in blue. Amino acids indicated in red represent deviations from conserved amino acids in canonical Ura9 sequences.


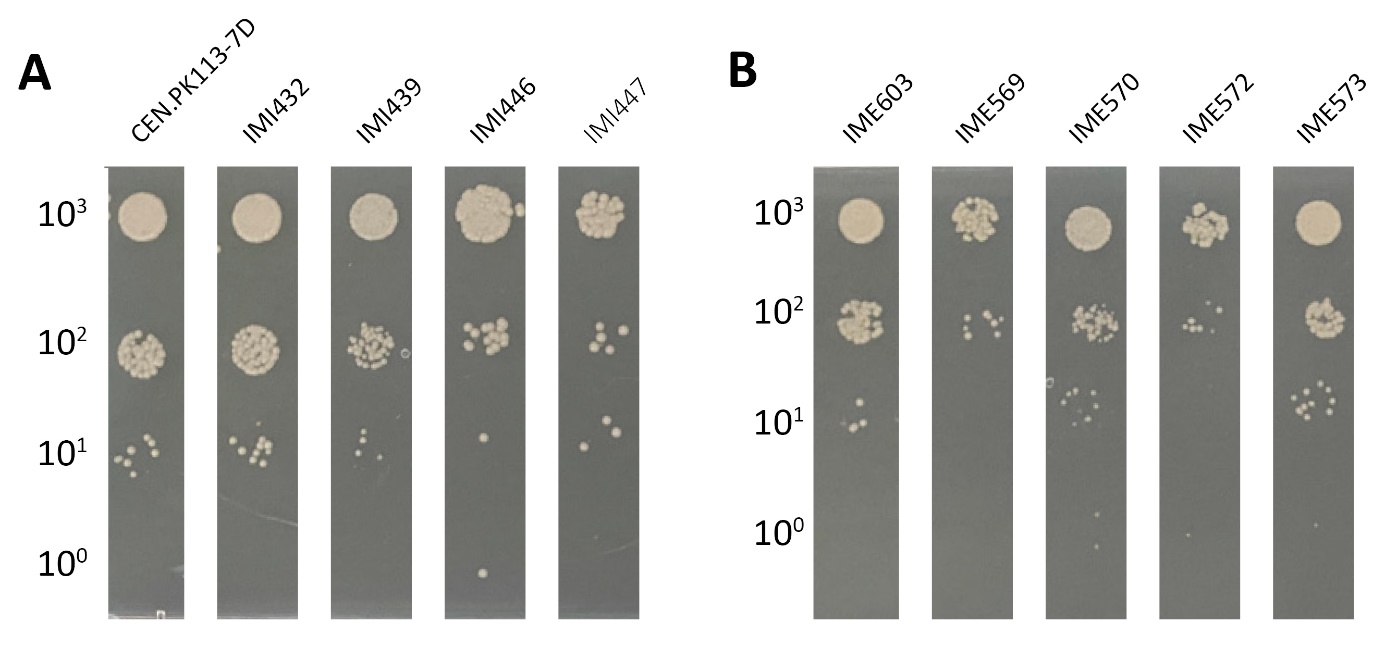


### Figure S3. Growth on non-fermentable carbon sources of *S. cerevisiae* strains expressing heterologous *URA9* genes.

The native *URA1* gene of *S. cerevisiae* (*ScURA*1) was either replaced by a single expression cassette for an *URA9* ortholog of *A. robustus* (Ar), *D. bruxellensis* (Db), *K. marxianus* (Km) or
*O. parapolymorpha* (Op), or expression cassettes introduced on multicopy plasmids (mc). Synthetic medium with ethanol and glycerol (SMEG) plates were incubated aerobically for 12 d at 20 °C**. A:**  Strains with single-copy DHOD genes: CEN.PK113-7D (Sc*URA1*; reference); IMI432 (*ura1Δ::Arura9*), IMI439 (*ura1Δ::DbURA9*), IMI446 (*ura1Δ::KmURA9*) and IMI447 (*ura1Δ::OpURA9*). **B:** Strains carrying multicopy plasmids: IME603 (mc*ScURA1*), IME569 (*ura1Δ* mc*Arura9*), IME570 (*ura1Δ* mc*DbURA9*), IME572 (*ura1Δ* mc*KmURA9*) and IME573 (*ura1Δ* mc*OpURA9*).


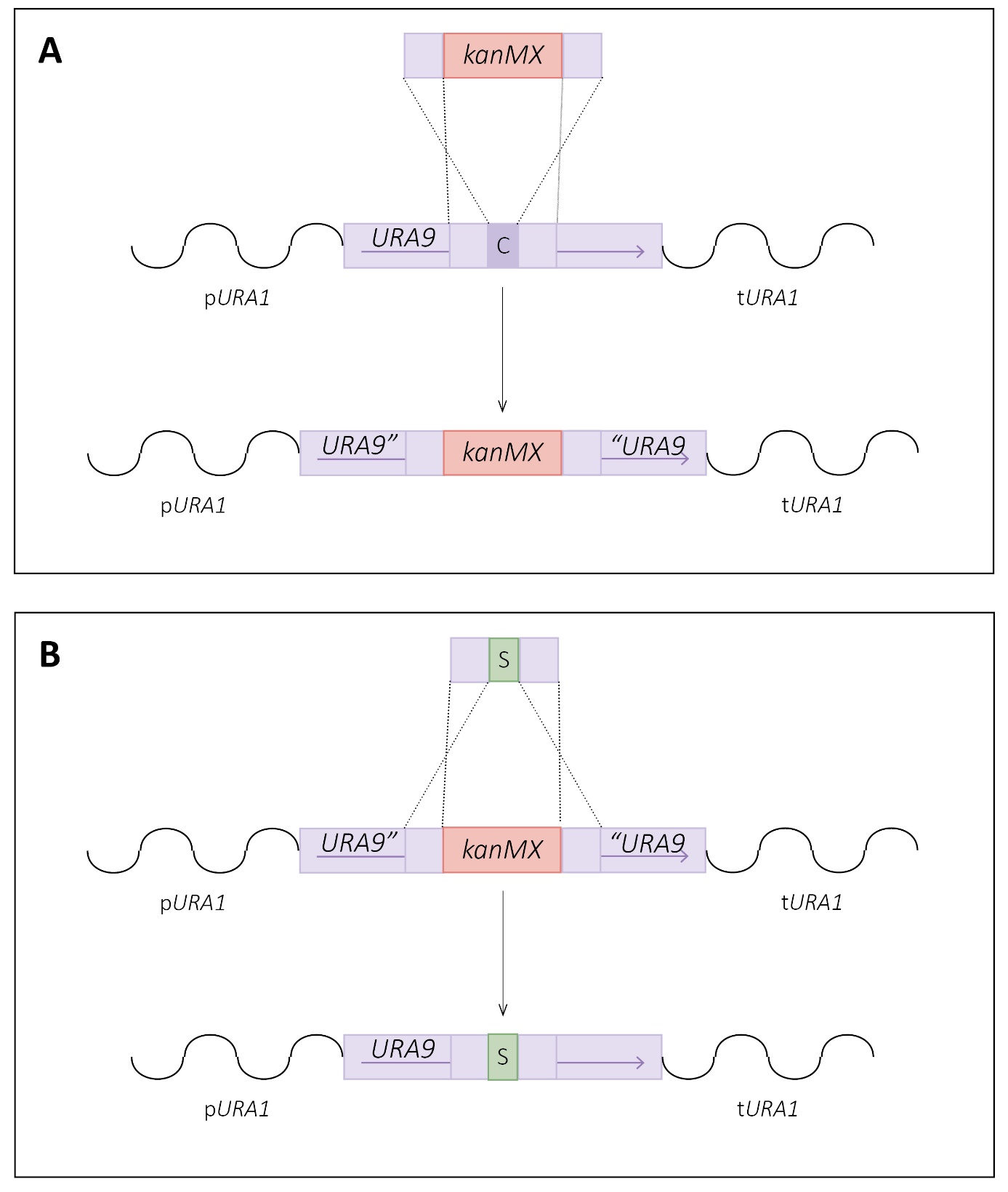


### Figure S4. Introduction of point mutations in *URA9* genes integrated in *S. cerevisiae*.

Point mutations were introduced in two rounds of transformation, **A:** In a first transformation, the codon of interest (purple C) in the integrated *URA9* gene (purple) was targeted with a gRNA to introduce a Cas9 induced double-strand break. A repair fragment encompassing a *kanMX* marker gene (red) flanked with 60 bp sequences homologous to sequences up- and downstream of the codon of interest was integrated at the site of the double-strand break, resulting in a strain in which the codon of interest was replaced by the *kanMX* marker. **B:** After verification of correct integration of the *kanMX* marker, it was targeted with a specific gRNA in a subsequent round of transformation. A 120 bp repair oligonucleotide homologous to the *URA9*-gene and carrying the mutated codon of interest (green S) was supplied. Homologous recombination resulted in a strain with a *URA9­* gene that contained a single-nucleotide mutation.

## Supporting Tables

### Table S1. Maximum specific growth rates under aerobic and anaerobic conditions for *URA1-* and *URA9*-expressing strains.

Specific growth rates were determined from at least five points in the exponential phase and presented as average ± mean deviation of measurements on duplicate cultures. Absence of exponential growth is indicated as n.g.; while n.a. indicates that the corresponding experiments was not performed.

| Strain | Relevant genotype | Parental strain | Maximum specific growth rate μ (h^-1^) | | | | | | |
| --- | --- | --- | --- | --- | --- | --- | --- | --- | --- |
|  |  |  | Aerobic | | | | Anaerobic | | |
|  |  |  | SMUD | | SMUD-ura | | SMUD | | SMUD-ura |
| Reference strains | | | | | | | | | |
| CEN.PK113-7D | *URA1* |  | 0.350±0.004 | | 0.357±0.003 | | 0.236±0.013 | | 0.247±0.011 |
| IMK824 | *ura1Δ* | IMX585 (SpCas9) | n.g | | 0.298±0.002 | | n.g. | | 0.256±0.009 |
| *URA9-*expressing strains | | | | | | | | | |
| IMI432 | *ura1Δ::Arura9* | IMX585 | 0.338±0.002 | | 0.349±0.000 | | 0.246±0.006 | | 0.254±.010 |
| IMI439 | *ura1Δ::DbURA9* | IMX585 | 0.342±0.004 | | 0.343±0.002 | | 0.206±0.006* | | 0.250±.007 |
| IMI452 | *ura1Δ::SjURA9* | IMX585 | 0.167±0.002 | | 0.171±0.000 | | 0.120±0.000 | | 0.143±0.000 |
| IMI462 | *ura1Δ::SjURA9* | IMX2600 (SpCas9) | 0.189±0.003 | | 0.192±0.002 | | 0.147±0.021 | | 0.153±0.023 |
| IMI446 | *ura1Δ::KmURA9* | IMX585 | 0.342±0.001 | | 0.348±0.001 | | n.g. | | 0.250±0.000 |
| IMI447 | *ura1Δ::OpURA9* | IMX585 | 0.352±.0.001 | | 0.348±0.003 | | n.g. | | 0.250±0.006 |
| DHOD genes expressed from a multicopy plasmid | | | | | | | | | |
| IME569 | 2mμ *Arura9* | IMK824 | 0.194±0.003 | | 0.273±0.004 | | n.a. | | n.a. |
| IME570 | 2mμ *DbURA9* | IMK824 | 0.208±0.002 | | 0.283±0.007 | | n.a. | | n.a. |
| IME571 | 2mμ *SjURA9* | IMK824 | 0.185±0.000 | | 0.195±0.005 | | n.a. | | n.a. |
| IME572 | 2mμ *KmURA9* | IMK824 | 0.267±0.004 | | 0.306±0.001 | | n.a. | | n.a. |
| IME573 | 2mμ *OpURA9* | IMK824 | 0.196±0.004 | | 0.273±0.004 | | n.a. | | n.a. |
| IME603 | 2mμ *ScURA1* | CEN.PK113-5 (*ura3-52*) | 0.242±0.005 | | 0.267±0.009 | | n.a. | | n.a. |
| Strains expressing mutated DHOD genes | | | | | | | | | |
| IMG005 | *ura1Δ::KmURA9*^S263C^ | IMI447 | 0.341±0.001 | | 0.345±0.004 | | n.g. | | 0.244±0.002 |
| IMG007 | *ura1Δ:: Arura9*^C168S^ | IMI432 | 0.322±0.005 | | 0.350±0.004 | | 0.240±0.003 | | 0.244±0.001 |
| IMG008 | *ura1Δ::SjURA9*^C265S^ | IMI452 | 0.086±0.000 | | 0.181±0.001 | | n.g. | | 0.127±0.002 |
| Selection strain | | | | | | | | | |
| IMS1206 | *ura1Δ* | IME571 | n.a. | 0.172±.001 | | n.a. | | n.a. | |
| IMS1167 | *ura1Δ::DbURA9* | IMI439 | n.a. | n.a. | | 0.219±0.002 | | 0.239±0.003 | |
| IMS1168 | *ura1Δ::DbURA9* | IMI439 | n.a. | n.a. | | 0.206±0.003 | | 0.226±.0.003 | |
| IMS1169 | *ura1Δ::DbURA9* | IMI439 | n.a. | n.a. | | 0.206±0.008 | | 0.232±0.001 | |
| IMS1170 | *ura1Δ::DbURA9* | IMI439 | n.a. | n.a. | | 0.191±0.012 | | 0.201±0.004 | |
| *Maximum specific growth rate was calculated from the second transfer (Figure S1). | | | | | | | | | |

Table S2. Sequence similarities of sequences in proteomes of (facultative) anaerobic fungi with those of the fumarate reductases Frd1 and Osm1 of *S. cerevisiae*.

Amino-acid sequences of Frd1 (GenBank accession number; EIW10990.1) and Osm1 (EIW09573.1) of *S. cerevisiae* CEN.PK113-7D (taxid 889517) were used as query. Results with an E-value below E^-50^ are shown. Localization of predicted proteins was predicted using WoLF PSORT [3] for fungal sequences and the highest scoring cellular component (mitochondria, cytosol, or other) is presented. The proteome of *P. finnis* yielded a partial protein sequence (ORX41846.1) whose localization could not be predicted (n.a.).

| Subject proteome | Resulting GenBank  accession | Query coverage  (%) | | E-value | | Identity  (%) | | Predicted localization |
| --- | --- | --- | --- | --- | --- | --- | --- | --- |
|  | Protein query | Frds1 | Osm1 | Frds1 | Osm1 | Frds1 | Osm1 |  |
| *A. robustus* | [ORX80473.1](https://www.ncbi.nlm.nih.gov/protein/ORX80473.1?report=genbank&log$=prottop&blast_rank=1&RID=D8RSVF8V016)  [ORX87251.1](https://www.ncbi.nlm.nih.gov/protein/ORX87251.1?report=genbank&log$=prottop&blast_rank=2&RID=D8RSVF8V016) | 97  98 | 91  95 | 6∙10^-99^  6∙10^-84^ | 3∙10^-102^  1∙10^-81^ | 40.99  35.86 | 41.54  33.58 | Cytosol  Other |
| *P. finnis* | [ORX59854.1](https://www.ncbi.nlm.nih.gov/protein/ORX59854.1?report=genbank&log$=prottop&blast_rank=1&RID=D8S4NZU5013)  [ORX41846.1](https://www.ncbi.nlm.nih.gov/protein/ORX41846.1?report=genbank&log$=prottop&blast_rank=2&RID=D8S4NZU5013) | 97  98 | 91  92 | 1∙10^-96^  2∙10^-88^ | 2∙10^-102^  3∙10^-90^ | 40.00  37.30 | 42.03  36.96 | Cytosol  n.a. |
| *N. californiae* | [ORY20775.1](https://www.ncbi.nlm.nih.gov/protein/ORY20775.1?report=genbank&log$=prottop&blast_rank=1&RID=D8S7PE0K01R)  [ORY34386.1](https://www.ncbi.nlm.nih.gov/protein/ORY34386.1?report=genbank&log$=prottop&blast_rank=2&RID=D8S7PE0K01R) | 98  97 | 91  92 | 9∙10^-100^  3∙10^-86^ | 1∙10^-103^  4∙10^-86^ | 40.93  36.86 | 41.85  35.98 | Cytosol  Other |
| *Sch. japonicus* | [XP_002171497.1](https://www.ncbi.nlm.nih.gov/protein/XP_002171497.1?report=genbank&log$=prottop&blast_rank=1&RID=D8SDB52201R) | 99 | 91 | 9∙10^-99^ | 2∙10^-96^ | 39.63 | 38.63 | Mitochondria |
| *D. bruxellensis* | [KAF6005858.1](https://www.ncbi.nlm.nih.gov/protein/KAF6005858.1?report=genbank&log$=prottop&blast_rank=1&RID=D92YFNZH013)  [VUG19043.1](https://www.ncbi.nlm.nih.gov/protein/VUG19043.1?report=genbank&log$=prottop&blast_rank=2&RID=FNAW9ECT013)  [XP_041138004.1](https://www.ncbi.nlm.nih.gov/protein/XP_041138004.1?report=genbank&log$=prottop&blast_rank=3&RID=FNAW9ECT013) | 97  97  97 | 92  98  92 | 8∙10^-104^  2∙10^-103^  4∙10^-103^ | 2∙10^-92^  5∙10^-94^  4∙10^-92^ | 41.61  41.61  41.61 | 39.09  38.13  38.89 | Mitochondria  Mitochondria  Mitochondria |

### Table S3. *S. cerevisiae* strains used in this study

| Strain | Relevant genotype | Parental strain | Reference |
| --- | --- | --- | --- |
| CEN.PK113-7D | *MAT***a** *TRP1 LEU2 HIS3 URA3* | - | [4] |
| CEN.PK113-5D | *MAT***a** *TRP1 LEU2 HIS3 ura3-52* | CEN.PK113-7D | [4] |
| IMX585 | *MAT***a** *TRP1 LEU2 HIS3* *URA3* c*an1Δ::Spcas9-natNT2* | CEN.PK113-7D | [5] |
| IMX2600 | *MAT***a** *TRP1 LEU2 HIS3* *URA3* c*an1Δ::Spcas9-natNT2* | CEN.PK113-7D | [6] |
| IMX581 | *MAT***a** *TRP1 LEU2 HIS3* *ura3-52* c*an1Δ::Spcas9-natNT2* | CEN.PK113-5D | [5] |
| IMK242 | *MAT***a** *TRP1 LEU2 HIS3* *URA3 rip1Δ::loxP*-*kanMX-loxP* | CEN.PK113-7D | [7] |
| IMK824 | *MAT***a** *TRP1 LEU2 HIS3* *URA3* c*an1Δ::Spcas9-natNT2 ura1Δ* | IMX585 | This study |
| IMK976 | *MAT***a** *TRP1 LEU2 HIS3* *ura3-52* c*an1Δ::Spcas9-natNT2 leu2Δ ura1Δ* | IMX581 | This study |
| IMI432 | *MAT***a** *TRP1 LEU2 HIS3* *URA3* c*an1Δ::Spcas9-natNT2 ura1Δ::Arura9* | IMX585 | This study |
| IMI439 | *MAT***a** *TRP1 LEU2 HIS3* *URA3* c*an1Δ::Spcas9-natNT2 ura1Δ::DbURA9* | IMX585 | This study |
| IMI446 | *MAT***a** *TRP1 LEU2 HIS3* *URA3* c*an1Δ::Spcas9-natNT2 ura1Δ::KmURA9* | IMX585 | This study |
| IMI447 | *MAT***a** *TRP1 LEU2 HIS3* *URA3* c*an1Δ::Spcas9-natNT2 ura1Δ::OpURA9* | IMX585 | This study |
| IMI452 | *MAT***a** *TRP1 LEU2 HIS3* *URA3* c*an1Δ::Spcas9-natNT2 ura1Δ::SjURA9* | IMX585 | This study |
| IMI462 | *MAT***a** *TRP1 LEU2 HIS3* *URA3* c*an1Δ::Spcas9-natNT2 ura1Δ::SjURA9* | IMX2600 | This study |
| IME569 | *MAT***a** *TRP1 LEU2 HIS3* *URA3* c*an1Δ::Spcas9-natNT2 ura1Δ* pUDE696 | IMK824 | This study |
| IME570 | *MAT***a** *TRP1 LEU2 HIS3* *URA3* c*an1Δ::Spcas9-natNT2 ura1Δ* pUDE738 | IMK824 | This study |
| IME571 | *MAT***a** *TRP1 LEU2 HIS3* *URA3* c*an1Δ::Spcas9-natNT2 ura1Δ* pUDE815 | IMK824 | This study |
| IME572 | *MAT***a** *TRP1 LEU2 HIS3* *URA3* c*an1Δ::Spcas9-natNT2 ura1Δ* pUDE756 | IMK824 | This study |
| IME573 | *MAT***a** *TRP1 LEU2 HIS3* *URA3* c*an1Δ::Spcas9-natNT2 ura1Δ* pUDE809 | IMK824 | This study |
| IME600 | *MAT***a** *TRP1 LEU2 HIS3* *ura3-52* c*an1Δ::cas9-SpnatNT2 leu2Δ ura1Δ* pUDC286 pUDE849 | IMK976 | This study |
| IME601 | *MAT***a** *TRP1 LEU2 HIS3* *ura3-52* c*an1Δ::Spcas9-natNT2 leu2Δ ura1Δ* pUDC286 pUDE1008 | IMK976 | This study |
| IME602 | *MAT***a** *TRP1 LEU2 HIS3* *ura3-52* c*an1Δ::Spcas9-natNT2 leu2Δ ura1Δ* pUDC286 pUDE1009 | IMK976 | This study |
| IME603 | *MAT***a** *TRP1 LEU2 HIS3* *URA3* c*an1Δ::cas9-SpnatNT2 ura1Δ* pUDE1069 | IIMK824 | This study |
| IME604 | *MAT***a** *TRP1 LEU2 HIS3* *ura3-52* c*an1Δ::Spcas9-natNT2 leu2Δ ura1Δ* pUDC286 pUDE1011 | IMK976 | This study |
| IMX2165 | *MAT***a** *TRP1 LEU2 HIS3* *URA3* c*an1Δ::Spcas9-natNT2 ura1Δ::SjURA9’-kanMX-‘SjURA9* | IMI452 | This study |
| IMX2203 | *MAT***a** *TRP1 LEU2 HIS3* *URA3* c*an1Δ::Spcas9-natNT2 ura1Δ::KmURA9’-kanMX-‘KmURA9* | IMI446 | This study |
| IMX2209 | *MAT***a** *TRP1 LEU2 HIS3* *URA3* c*an1Δ::Spcas9-natNT2 ura1Δ::Arura9’-kanMX-‘Arura9* | IMI432 | This study |
| IMG005 | *MAT***a** *TRP1 LEU2 HIS3* *URA3* c*an1Δ::Spcas9-natNT2 ura1Δ::KmURA9*^S263C^ | IMX2203 | This study |
| IMG007 | *MAT***a** *TRP1 LEU2 HIS3* *URA3* c*an1Δ::Spcas9-natNT2 ura1Δ::Arura9*^C168S^ | IMX2209 | This study |
| IMG008 | *MAT***a** *TRP1 LEU2 HIS3* *URA3* c*an1Δ::Spcas9-natNT2 ura1Δ::SjURA9*^C265S^ | IMX2165 | This study |
| IMS1167 | Single colony isolate 1.1 of IMI439 anaerobically evolved for growth on SMUD | IMI439 | This study |
| IMS1168 | Single colony isolate of 1.2 IMI439 anaerobically evolved for growth on SMUD | IMI439 | This study |
| IMS1169 | Single colony isolate of 2.1 IMI439 anaerobically evolved for growth on SMUD | IMI439 | This study |
| IMS1170 | Single colony isolate of 2.2 IMI439 anaerobically evolved for growth on SMUD | IMI439 | This study |
| IMS1206 | Single colony isolate of IME573 selected for plasmid removal | IME573 | This study |

### Table S4. Dihydroorotate activities of ArUra9 with various electron acceptors, measured in cell extracts of *S. cerevisiae* IME569.

Activities were determined in 0.1 M potassium phosphate buffer (pH 7.5) at 30 °C, by measuring orotate formation at 300 nm. Reactions were started with the addition of dihydroorotate. Activities represent the average and mean deviation of results obtained with two separately prepared cell extracts. Reduction potentials were retrieved from Schafer and Buettner [2]. PMS; phenazine methosulfate, DHAP; dihydroxyacetone phosphate.

| Electron acceptor | Reduction potential  (V) | Activity  (U∙mg_protein_^-1^) |
| --- | --- | --- |
| - | - | 0.008±0.001 |
| DHAP | -0.29 | 0.008±0.001 |
| FAD | -0.219 | 0.148±0.032 |
| FMN | -0.219 | 0.126±0.012 |
| Acetaldehyde | -0.197 | 0.014±0.008 |
| Pyruvate | -0.183 | 0.008±0.002 |
| Oxaloacetate | -0.166 | 0.008±0.001 |

### Table S5. Oligonucleotides used in this study

| Number | Sequence 5’ → 3’ |
| --- | --- |
| 580 | GAATGTAAGCGTGACATAAC |
| 1552 | TAGGATGAGTAGCAGCACGTTC |
| 1720 | CAATTCAGCGCAGTCACG |
| 1935 | ATCCGTACTCCTGATGATGC |
| 2897 | CCAGTGCTGCAATGATACC |
| 2898 | TATTGACGCCGGGCAAGAG |
| 3452 | GACGGACTTCAGCAATGGCATTC |
| 4369 | TGGGCATGTACGGGTTACAG |
| 5719 | CGAATACCGCTTCCACAAAC |
| 5720 | GTTTGTGGAAGCGGTATTCG |
| 6005 | GATCATTTATCTTTCACTGCGGAGAAG |
| 6006 | GTTTTAGAGCTAGAAATAGCAAGTTAAAATAAGGCTAGTC |
| 7823 | ATCCGTCGAAACTAAGTTCTGG |
| 7998 | AAGCTTATCGATACCGTCGACC |
| 9595 | TGTAATATCTTAATGCTAAA |
| 11334 | TGCGCATGTTTCGGCGTTCGAAACTTCTCCGCAGTGAAAGATAAATGATCGGTACAGAATTACAAAAAGAGTTTTAGAGCTAGAAATAGCAAGTTAAAATAAGGCTAGTCCGTTATCAAC |
| 11335 | GTTGATAACGGACTAGCCTTATTTTAACTTGCTATTTCTAGCTCTAAAACTCTTTTTGTAATTCTGTACCGATCATTTATCTTTCACTGCGGAGAAGTTTCGAACGCCGAAACATGCGCA |
| 11336 | TAGACGTTTCAAATAGATATACACAATTACTCAAAAAAAAATTGAACTTCCGTACCAAACTTATCGGAATTTGAAGAACTATGATGGGACAAGTTATGGAAGAGTGTTAAGATTCGTATG |
| 11337 | CATACGAATCTTAACACTCTTCCATAACTTGTCCCATCATAGTTCTTCAAATTCCGATAAGTTTGGTACGGAAGTTCAATTTTTTTTTGAGTAATTGTGTATATCTATTTGAAACGTCTA |
| 11338 | TTTTCTTCACCGGGCTTTGC |
| 11353 | GATGCCAATTGCGAATGCACTC |
| 11634 | GTGAGCAAGGGCGAGGAG |
| 11798 | CGACGGATTCTAGAACTAGTGGGTCTCATATGGTTTTGATCTC |
| 11799 | GAATGCCTTAGTCATGCCAACCCGGTCTCAGGATTTAAGCCTTGTGTTC |
| 11802 | TAGACGTTTCAAATAGATATACACAATTACTCAAAAAAAAATTGAACTTCCGTACCAAACCGCGTGTGGAAGAACGATTACAACAGG |
| 11803 | CATACGAATCTTAACACTCTTCCATAACTTGTCCCATCATAGTTCTTCAAATTCCGATAAGCTGGAGCTCAGTTTATCATTATC |
| 11808 | CAGCGTAAGAACCCAATTCC |
| 12367 | AGAACTTAGTTTCGACGGATGCGGTACCATGTCTTTCAAC |
| 12368 | TCGACGGTATCGATAAGCTTTGCTCCGAGCTCTTAGTTC |
| 12375 | CACGATCTTCGGCATTCGCCGAAAATTTTTCAAGATGCCCATCACCAAAAAATAAAAAAAGCGATCGCGTGTGGAAGAAC |
| 12376 | CATACGAATCTTAACACTCTTCCATAACTTGTCCCATCATAGTTCTTCAAATTCCGATAAGCTGGAGCTCAGTTTATCATTATC |
| 12665 | GCGTTTGACACACGTTCGATGCTACCTGTTCCATCAGTGTTTATGCCATTTGAGCCCTGGACACACCGAGATTCATCAAC |
| 12735 | AGGCAACTTGACACCAAGAG |
| 12743 | TGCGCATGTTTCGGCGTTCGAAACTTCTCCGCAGTGAAAGATAAATGATCACCGGATTCAGTCGTCACTCAGTTTTAGAGCTAGAAATAGCAAGTTAAAATAAG |
| 12744 | TGCGCATGTTTCGGCGTTCGAAACTTCTCCGCAGTGAAAGATAAATGATCAGACCTGAAATCAAAGTTATGTTTTAGAGCTAGAAATAGCAAGTTAAAATAAG |
| 12842 | GGAGCAGATGGACAAGAAAC |
| 13277 | TGCGCATGTTTCGGCGTTCGAAACTTCTCCGCAGTGAAAGATAAATGATCCTTTTGGAAGTTAAAGACATGTTTTAGAGCTAGAAATAGCAAGTTAAAATAAG |
| 13278 | TTGAACATAAATAATATCACTCTAACACTTATTAGGAAACCGAAAGGAGCAATAACAAACGTTTGTTTTTCGTCCTCGAAAACTACATCTTTATTGTCTATTTGTTTCACCCTTATTTTG |
| 13279 | CAAAATAAGGGTGAAACAAATAGACAATAAAGATGTAGTTTTCGAGGACGAAAAACAAACGTTTGTTATTGCTCCTTTCGGTTTCCTAATAAGTGTTAGAGTGATATTATTTATGTTCAA |
| 13674 | TGCGCATGTTTCGGCGTTCGAAACTTCTCCGCAGTGAAAGATAAATGATCGTTTCTTGTCCAAACTTGCCGTTTTAGAGCTAGAAATAGCAAGTTAAAATAAG |
| 13715 | TGCGCATGTTTCGGCGTTCGAAACTTCTCCGCAGTGAAAGATAAATGATCGTTTCTTGCCCAAACATTTCGTTTTAGAGCTAGAAATAGCAAGTTAAAATAAG |
| 13800 | AGAACTTAGTTTCGACGGATCCATGTCCTCTACTTTTAAGTCCGC |
| 13801 | TCGACGGTATCGATAAGCTTGCTCTTAAGCAGATTCGGTGCC |
| 13802 | AACTTCAACGTAGGCGTAACC |
| 13811 | CGCATATACCTTTTTCAACTGAAAAATTGGGAGAAAAAGGAAAGGTGAGAGCGCCGGAACGATATTTGTACATAAACTTTATAAATGAAATTCATAATAGAAACGACACGAAATTACAAA |
| 13812 | TTTGTAATTTCGTGTCGTTTCTATTATGAATTTCATTTATAAAGTTTATGTACAAATATCGTTCCGGCGCTCTCACCTTTCCTTTTTCTCCCAATTTTTCAGTTGAAAAAGGTATATGCG |
| 14345 | AATTGAAGGCGGACCACTACC |
| 14439 | GGGCGCTATCGCACAGAATC |
| 14440 | ACTGAAGAGGAGGTCGACTACG |
| 14480 | TTGTTCGTACTTCCTTCTGAGCAG |
| 14708 | AGAACTTAGTTTCGACGGATGTAGTTGCATGTCCATTGGAAAG |
| 14709 | TCGACGGTATCGATAAGCTTTCTTATTTGACGTCTTCTCC |
| 14710 | AGAACTTAGTTTCGACGGATATGTTTTCCCGAACAGGTCTC |
| 14711 | TCGACGGTATCGATAAGCTTCTATTTATCGTCCTCGCCAACTAC |
| 14759 | GAACCGGTATTCTTCTCTTGG |
| 14760 | CCAATTGCGAATGCACTCAC |
| 14872 | CCCACGCATGTATCTATCTC |
| 14873 | ACTTGCCAGGTTTGAGAG |
| 14873 | ACTTGCCAGGTTTGAGAG |
| 14874 | CTTGGCAATTCTTCCTTAGC |
| 14875 | CGATGATTGGTGGCTTTC |
| 14876 | AGATTGAACGCTGACGAAAC |
| 14877 | TCGGTGAAGAACACAAGG |
| 14878 | CTCTTGGGTAGTTGTCAGAG |
| 15067 | TGCGCATGTTTCGGCGTTCGAAACTTCTCCGCAGTGAAAGATAAATGATCTGATTATAATACCATTTAGGGTTTTAGAGCTAGAAATAGCAAGTTAAAATAAGGCTAGTCCGTTATCAAC |
| 15597 | TTAAGCCTTGTGTTCTTCACCGATGATG |
| 15598 | ATGGTTTTGATCTCTGACAACTAC |
| 15645 | TGAAGTGGTGGCCTAACTACGG |
| 15651 | AATATTTTAGTAGCTCGTTACAGTCCG |
| 15834 | ACGTTTTGGGTGTTAAGGAATTGGGTTCTTACGCTGACTTCATCGTTATCAACGTTTCTTCTCCAAACTTGCCAGGTTTGAGAGCTTTGCAAGAAAAGGACACTTTGAAGAACTTGTTGG |
| 15835 | CCAACAAGTTCTTCAAAGTGTCCTTTTCTTGCAAAGCTCTCAAACCTGGCAAGTTTGGAGAAGAAACGTTGATAACGATGAAGTCAGCGTAAGAACCCAATTCCTTAACACCCAAAACGT |
| 15836 | TGCGCATGTTTCGGCGTTCGAAACTTCTCCGCAGTGAAAGATAAATGATCGTTTCCTCTCCAAACACACCGTTTTAGAGCTAGAAATAGCAAGTTAAAATAAG |
| 15837 | CAGGACTACTTGAAAGGTGTTGAAAAATTCCAGAAATATGCAGATGTGCTAGTGATTAACCATGGGTAAGGAAAAGACTC |
| 15838 | GCGACCACCTGACTCAAAAGATTAGTTAAAATCGATTCCTTCTGCAAGCCTCTGAGTCCACAAGTTCTTGAAAACAAGAATC |
| 15847 | GCAACAACTTTGTTCAACAGGGGAACCAAAGTATCCTTAGATTGCAAGTTGCAGACACCACATGGGTAAGGAAAAGACTC |
| 15848 | GAGGATTTCTTGAAAGGTGTTAGAAAATTCGGTAACTTCGCCGATATCTTGGTTGTTAATCAAGTTCTTGAAAACAAGAATC |
| 15851 | GACGACTACGTTTTGGGTGTTAAGGAATTGGGTTCTTACGCTGACTTCATCGTTATCAACCATGGGTAAGGAAAAGACTC |
| 15852 | TTGATAACAGCTTCCAACAAGTTCTTCAAAGTGTCCTTTTCTTGCAAAGCTCTCAAACCTCAAGTTCTTGAAAACAAGAATC |
| 16360 | GGTCTCATATGTTGTCTTTGAGACAATCTATCAGATTCTTCAAGCCAGCTACTAGAACTTTGTGTTCTTCTAGATACTTGTTGGGTTCTTGAGACC |
| 16361 | GGTCTCAAGAACCCAACAAGTATCTAGAAGAACACAAAGTTCTAGTAGCTGGCTTGAAGAATCTGATAGATTGTCTCAAAGACAACATATGAGACC |
| 16368 | CTTGAAAGGTGTTGAAAAATTCCAGAAATATGCAGATGTGCTAGTGATTAACGTTTCCTGTCCAAACACACCTGGACTCAGAGGCTTGCAGAAGGAATCGATTTTAACTAATCTTTTGAG |
| 16369 | CTCAAAAGATTAGTTAAAATCGATTCCTTCTGCAAGCCTCTGAGTCCAGGTGTGTTTGGACAGGAAACGTTAATCACTAGCACATCTGCATATTTCTGGAATTTTTCAACACCTTTCAAG |
| 16370 | ACAGGGGAACCAAAGTATCCTTAGATTGCAAGTTGCAGACACCAGAAATGTTTGGGGAAGAAACATTAACAACCAAGATATCGGCGAAGTTACCGAATTTTCTAACACCTTTCAAGAAAT |
| 16371 | ATTTCTTGAAAGGTGTTAGAAAATTCGGTAACTTCGCCGATATCTTGGTTGTTAATGTTTCTTCCCCAAACATTTCTGGTGTCTGCAACTTGCAATCTAAGGATACTTTGGTTCCCCTGT |
| 16436 | AGAACGGTGACGAAGTACAG |
| 16438 | GGTAACTTCGCCGATATCTTGG |
| 16439 | CAAGTCGAGGTCTGCGATTC |
| 16441 | AGAACGGTGACGAAGTACAG |
| 16443 | CTTGAGCGACGAGATTCTTG |
| 16443 | CTTGAGCGACGAGATTCTTG |
| 16445 | GAGTCACAGATCCGATTTCC |
| 16449 | CCATTGCCAAGGCTGCGAAG |
| 16451 | CAGCCTGGTTGATGAGGTTC |
| 16454 | GCACCAGCTCTAGCAAATTC |
| 16455 | CGGTCAAATCTGGACCAATC |
| 16456 | GTTACCTGGTTGTGGTTCTG |
| 16458 | TACGCCTACGTTGAAGTTGG |
| 17291 | CTTCCGAAAATGCAACGCGAGGCCGCAAATTAAAGCCTTCGAG |
| 17292 | CTCGCGTTGCATTTTCGGAAG |
| 17293 | CTTTTTACGGTTCCTGGCCTCACGCCCTCCAACGAAGC |
| 17294 | GCACGTGACTGCGCTGAATTGCAAAAGCTGGAGCTCAGTTTATC |
| 17296 | AGCTCCTCGCCCTTGCTCACTTTATCGTCCTCGCCAACTAC |
| 17298 | AGCTCCTCGCCCTTGCTCACAGCCTTGTGTTCTTCACC |
| 17299 | AGCTCCTCGCCCTTGCTCACGTTCTTTTCGTCGTCAGAACC |
| 17300 | AGCTCCTCGCCCTTGCTCACAGCAGATTCGGTGCC |
| 17683 | GCTTACATTCACGCCCTCCC |
| 17697 | AGAACTTAGTTTCGACGGATCATGACAGCCAGTTTAACTACC |
| 17698 | TCGACGGTATCGATAAGCTTTTAAATGCTGTTCAACTTCCC |

### Table S6. Plasmids used in this study

| Plasmid | Relevant characteristics | Reference |
| --- | --- | --- |
| pMEL13 | AmpR *kanMX* gRNA-*CAN1*.Y 2μm | [5] |
| pROS12 | AmpR *hygB* gRNA-*CAN1*.Y 2μm gRNA-*ADE2*.Y | [5] |
| pROS13 | AmpR *kanMX* gRNA-*CAN1*.Y 2μm gRNA-*ADE2*.Y | [5] |
| pROS14 | AmpR *KlLEU2* gRNA-*CAN1*.Y 2μm gRNA-*ADE2*.Y | [5] |
| pYTK002 | ConLS CamR (type 1) | [8] |
| pYTK009 | *pTDH3* CamR (type 2) | [8] |
| pYTK033 | *tADH1* CamR (type 4) | [8] |
| pYTK046 | mRuby2 CamR (type 3b) | [8] |
| pYTK047 | *E. coli* GFP-dropout (type 234r) | [8] |
| pYTK067 | ConRI (type 5) | [8] |
| pYTK074 | *URA3* (type 6) | [8] |
| pYTK081 | CEN6/ARS (type 7) | [8] |
| pYTK089 | AmpR-ColE (type 8a) | [8] |
| pUDC071 | AmpR *URA3* p*ARO10-*eGFP-t*CYC1* 2μm | [9] |
| pUDC286 | AmpR *URA3* p*TDH3-*(*ScCOX4* MTS)-mRuby2*-*t*ADH1* 2μm | This study |
| pUD538 | CamR *URA3* GFP-dropout CEN6/ARS | This study |
| pUD650 | AmpR *Arura9* | Ordered from GeneArt |
| pUD707 | AmpR *DbURA9* | Ordered from GeneArt |
| pUD764 | AmpR *SjURA9* | Ordered from GeneArt |
| pUDR348 | AmpR *kanMX* gRNA-*URA1* 2μm gRNA-*URA1* | This study |
| pUDR499 | AmpR *kanMX* gRNA-*Arura9-* 2μm gRNA-*Arura9* | This study |
| pUDR501 | AmpR *kanMX* gRNA-*SjURA9-* 2μm gRNA-*SjURA9* | This study |
| pUDR602 | AmpR *kanMX* gRNA-*KmURA9-* 2μm gRNA-*KmURA9* | This study |
| pUDR605 | AmpR *hugB* gRNA-*kanMX-* 2μm gRNA-*kanMX* | This study |
| pUDR721 | AmpR *kanMX* gRNA-*LEU2* 2μm gRNA-*URA11* | This study |
| pUDE63 | AmpR *URA3* p*TDH3*-*pgmB*-t*CYC1* 2μm | [10] |
| pUDE696 | AmpR *URA3* p*TDH3*-*Arura9*-t*CYC1* 2μm | This study |
| pUDE738 | AmpR *URA3* p*TDH3*-*DbURA9*-t*CYC1* 2μm | This study |
| pUDE756 | AmpR *URA3* p*TDH3*-*KmURA9*-t*CYC1* 2μm | This study |
| pUDE809 | AmpR *URA3* p*TDH3*-*OpURA9*-t*CYC1* 2μm | This study |
| pUDE815 | AmpR *URA3* p*TDH3*-*SjURA9*-t*CYC1* 2μm | This study |
| pUDE849 | AmpR *LEU2* p*TDH3*-*Arura9*-*eGFP*-t*CYC1* 2μm | This study |
| pUDE1008 | AmpR *LEU2* p*TDH3*-*DbURA9*-*eGFP*-t*CYC1* 2μm | This study |
| pUDE1009 | AmpR *LEU2* p*TDH3*-*SjURA9*-*eGFP*-t*CYC1* 2μm | This study |
| pUDE1011 | AmpR *LEU2* p*TDH3*-*OpURA9*-*eGFP*-t*CYC1* 2μm | This study |
| pUDE1069 | AmpR *URA3* p*TDH3*-*ScURA1*-t*CYC1* 2μm | This study |

# References

1. Sousa FM, Refojo PN, Pereira MM. Investigating the amino acid sequences of membrane bound dihydroorotate:quinone oxidoreductases (DHOQOs): Structural and functional implications. Biochim Biophys Acta - Bioenerg. 2021;1862:148321.

2. Schafer FQ, Buettner GR. Redox state and redox environment in biology. In: Forman HJ, Fukuto J, Torres M, editors. Signal transduction by reactive oxygen and nitrogen species: pathways and chemical principles. Dordrecht: Springer Netherlands; 2003. p. 1–14.

3. Horton P, Park KJ, Obayashi T, Fujita N, Harada H, Adams-Collier CJ, Nakai K. WoLF PSORT: Protein localization predictor. Nucleic Acids Res. 2007;35:585–7.

4. Entian KD, Kötter P. Yeast genetic strain and plasmid collections. In: Stansfield, I. , and Stark MJR, editor. Methods in microbiology: Yeast gene analysis. Amsterdam: Academic Press; 2007. p. 629–66.

5. Mans R, van Rossum HM, Wijsman M, Backx A, Kuijpers NGA, van den Broek M, Daran-Lapujade P, Pronk JT, van Maris AJA, Daran J-MG. CRISPR/Cas9: a molecular Swiss army knife for simultaneous introduction of multiple genetic modifications in *Saccharomyces cerevisiae*. FEMS Yeast Res. 2015;15:fov004.

6. Bouwknegt J, Wiersma SJ, Ortiz-Merino RA, Doornnebal ESR, Buitenhuis P, Giera M, Muller C, Pronk JT. A squalene-hopene cyclase in *Schizosaccharomyces japonicus* represents a eukaryotic adaptation to sterol‐independent anaerobic growth. BioRXiv [preprint]. 2021;

7. Perez-Samper G, Cerulus B, Jariani A, Vermeersch L, Simancas NB, Bisschops MMM, van den Brink J, Solis-Escalante D, Gallone B, De Maeyer D, van Bael E, Wenseleers T, Michiels J, Marchal K, Daran-Lapujade P, Verstrepen KJ. The crabtree effect shapes the *Saccharomyces cerevisiae* lag phase during the switch between different carbon sources. MBio. 2018;9:1–18.

8. Lee ME, DeLoache WC, Cervantes B, Dueber JE. A highly characterized yeast toolkit for modular, multipart assembly. ACS Synth Biol. 2015;4:975–86.

9. Romagnoli G, Knijnenburg TA, Litti G, Louis EJ, Pronk JT, Daran J-M. Deletion of the *Saccharomyces cerevisiae ARO8* gene, encoding an aromatic amino acid transaminase, enhancesphenylethanol production from glucose. Yeast. 2015;32:29–45.

10. de Kok S, Yilmaz D, Suir E, Pronk JT, Daran JM, van Maris AJA. Increasing free-energy (ATP) conservation in maltose-grown *Saccharomyces cerevisiae* by expression of a heterologous maltose phosphorylase. Metab Eng. 2011;13:518–26.
