# Supplementary material for: Class-II dihydroorotate dehydrogenases from three phylogenetically distant fungi support anaerobic pyrimidine biosynthesis
Source: Fungal Biol Biotechnol. 2021 Oct 16;8:10. doi: 10.1186/s40694-021-00117-4 (PMC8520639; doi:10.1186/s40694-021-00117-4)
Supplement: Supplementary file 6 — Additional file 6. Plasmid and strain construction. [file 40694_2021_117_MOESM6_ESM.docx]

### Plasmid and strain construction

### Plasmid construction

Optimization of coding sequences for expression in *S. cerevisiae* was performed by the GeneOptimizer tool (GeneArt; [1]). To construct plasmids harboring expression cassettes with different dihydroorotate dehydrogenase genes, codon-optimized *URA9* sequences (Additonal file 6) from *A. robustus* Sp. S4 (*Arura9,* UniProtKB accession number A0A1Y1XN91),
*D. bruxellensis* CBS72 (*DbURA9*, A2VBP6) and *Sch. japonicus* yFS275 (*SjURA9,* B6JXQ5) were amplified from pUD650, pUD707 and pUD764 (Additional file 3; Table S6) using oligonucleotides (Additional file 3; Table S5) 11798/11799, 12367/12368 and 13800/13801, respectively. In addition, *URA9*-genes from *K. marxianus* CBS6556 and
*O. parapolymorpha* CBS11895, and *URA1* from *S. cerevisiae* CEN.PK113-7D were amplified from genomic DNA using oligonucleotide primers 14708/14709, 14710/14711 and 17697/17698, respectively, resulting in *KmURA9*, *OpURA9* and *ScURA1,* respectively. These primers introduced 20 bp flanks with the pUDE63 plasmid to enable insertion of the coding sequences between the *pTDH3* and *tADH1* sequences by homologous recombination. The *pTDH3* and *tADH1* containing backbone was amplified from pUDE63 using primers 7823 and 7998. Assembly of this backbone with *Arura9*, *DbURA9, SjURA9, KmURA9, OpURA9* and *ScURA1* resulted in pUDE696, pUDE738, pUDE815, pUDE756, pUDE809 and pUDE1069, respectively. Correct assembly was confirmed by diagnostic PCR using one primer binding inside the *URA9/URA1* genes (primers 11808, 12842, 13802, 16436, 16449 and 12735 for *Arura9, DbURA9, SjURA9, KmURA9, OpURA9* and *ScURA1*, respectively) and one in the pUDE63 backbone (2897 for pUDE696*,* pUDE756, pUDE809 and 2898 for pUDE738, pUDE815, pUDE1069).

Plasmids containing gRNA-sequences to target genes for Cas9-mediated genome editing were constructed as follows. For the construction of a plasmid targeting *S. cerevisiae* *URA1* (pUDR348)*,* a backbone fragment (that contained an origin of replication for *E. coli*, an AmpR-marker and a *kanMX*-marker for use in yeast) and a 2μm-fragment flanked by *URA1*-targeting sequences were amplified from the pMEL13 plasmid using primer pairs 6005/6006 and 11334/11335, respectively. pUDR348 resulted from *in vivo* assembly of these two fragments in yeast and subsequent recovery from *S. cerevisiae* IMK824 (see Yeast strain construction, below) with the Zymoprep Yeast Plasmid Miniprep II kit (Zymo Research, Irvine, CA), after which it was transformed to *E. coli* for amplification and storage. *LEU2-* and *URA1-*targeting plasmid pUDR721 was constructed by Gibson Assembly of a backbone fragment amplified from pROS13 (containing the *E. coli* origin of replication, AmpR-marker and *kanMX*-marker sequence) using primer 6005 and a 2μm cassette flanked by gRNA-sequences amplified from pROS13 using primer pair 11334/15067. gRNA expression plasmids targeting heterologous genes *Arura9, SjURA9* and *KmURA9* were assembled from a pROS13 backbone (amplified using primer 6005) and a 2μm fragment with gRNA-sequences amplified with oligonucleotide 13674, 13715 or 15836, respectively, using pROS13 as template. This resulted in plasmids pUDR499, pUDR501 and pUDR602, respectively. pUDR605, which targets the *kanMX*-marker, was constructed by assembly of a pROS12 backbone and a 2μm-fragment with gRNA-sequences that were both amplified from pROS12 using primer 6005 and 12743, respectively.

pUDC286, a plasmid harboring an expression cassette for mRuby2 fused to a *COX4­*-MTS (mitochondrial targeting sequence), was constructed by Golden Gate Assembly. First, backbone plasmid pUD538 was constructed by assembly of type 1 part ConSL (pYKT002), 234r part *E. coli*  GFP drop-out (pYKT047), type 5, 6, 7 and 8a parts ConRI (pYKT067), *URA3* (pYKT074), CEN6/ARS (pYKT081) and AmpR-ColE (pYKT089), respectively [2]. Then, the *COX4-*MTS was made by annealing two single stranded oligonucleotides (16360/16361; Additional file 3; Table S5), yielding a type 3a part. The type 2, 3b and 4 parts were derived from plasmids (pYKT009, pYKT046 and pYKT033 respectively) available from the Yeast Toolkit [2]. Digestion and Golden Gate assembly as described by Lee et al. (2015) of the backbone of pUD538, the annealed *COX4*-MTS cassette, *pTDH3* from pYKT009, mRuby2 from pYKT046 and *tADH1* from pYKT033 resulted in pUDC286.

eGFP-fused-*URA9* expression plasmids with a *LEU2*-marker were assembled from the *LEU2­*-containing backbone of pROS14, a fragment containing the *THD3* promoter and a *URA9* gene without stop codon, an eGFP-*tCYC1* cassette and a 2μm fragment. The backbone and 2μm fragment were both obtained by PCR using pROS14 as template with primer pairs 1720/9395 and 17292/17293, respectively, that introduced a 20 bp overlap with the pROS14 backbone. An eGFP-*tCYC1* cassette was amplified from pUDC071 with primer 11634 and 17291, introducing a 20 bp overlap with the 2μm cassette. Primers for construction of *pTDH3-URA9* cassettes were designed to introduce a 20 bp overlap with the pROS14 backbone and eGFP cassette. For construction of the cassettes with *Arura9*, *DbURA9, SjURA9* and *OpURA9* respectively, pUDE696, pUDE738, pUDE815 and pUDE809 were used as template for PCR using oligonucleotide 17294 (binding *pTDH3* and introducing an overlap with pROS14) and oligonucleotides 17298, 17299, 172300 and 17296, respectively. Subsequent Gibson Assembly of the four fragments yielded plasmids pUDE849, pUDE1008, pUDE1009 and pUDE1011 for eGFP fused to *Arura9*, *DbURA9, SjURA9* and *OpURA9*, respectively. Correct assembly was verified by diagnostic PCR of from *tCYC1* to the 2μm fragment (primers 17683/5720), from the 2μm fragment to the backbone (primers 5719/2898), from the backbone to *pTDH3* (primers 3452/4369) and from the *URA9* gene to the eGFP-*tCYC1* sequence (using primer 580 binding *tCYC1* and 14877, 14876, 16438 or 16449 binding in the *Arura9*, *DbURA9*, *SjURA9* and *OpURA9* respectively).

### Yeast strain construction

*URA1* was deleted in the Cas9-expressing *S. cerevisiae* strain IMX585 by co-transformation of the pMEL13 backbone-fragment and 2μm cassette flanked by gRNA-sequences targeting *URA1*, that were described for the construction of plasmid pUDR348 in the previous paragraph, together with a double-stranded DNA fragment (annealed oligonucleotides 11336 and 11337), consisting of 60 bp sequences up- and downstream of *URA1* as described by [3]. This resulted in strain IMK824. To simultaneously delete *LEU2* and *URA1*, the Cas9-expressing strain IMX581 was transformed with the annealed oligonucleotide pair 13811/13812 and pUDR721, yielding strain IMK976. Deletions of *URA1* and *LEU2* were confirmed by diagnostic PCRs with primer pairs 11338/11353 and 14439/14440, respectively. Expression cassettes for *Arura9, DbURA9, SjURA9, KmURA9* and *OpURA9* were amplified from pUDE696, pUDE738, pUDE815, pUDE756 and pUDE809, respectively, using oligonucleotides 11802 and 11803, which introduced 60 bp sequences homologous to those flanking the *URA1* locus. Co-transformation of strain IMX585 with these expression cassettes and the *URA1*-targeting gRNA expression plasmid pUDR348 then yielded *S. cerevisiae* strains IMI432 (*ura1Δ::Arura9*), IMI439 (*ura1Δ::DbURA9*), IMI452 (*ura1Δ::SjURA9*), IMI446 (*ura1Δ::KmURA9*) and IMI447 (*ura1Δ::OpURA9*). The *SjURA9* expression cassette was similarly integrated in the *URA1* locus of the Cas9-expressing strain IMX2600 [4], resulting in strain IMI462. Correct integration was confirmed by diagnostic PCR with primers 11338 and 11353.

For expression of multiple copies of heterologous *URA9* genes in *S. cerevisiae*, the *ura1Δ* strain IMK824 was transformed with multi-copy plasmids (Additional file 3; Table S6) carrying expression cassettes of *URA9* between a *TDH3* promoter and *ADH1* terminator. Transformation with the 2μ plasmids pUDE596 (*Arura9*) and, pUDE738 (*DbURA9*), pUDE815 (*SjURA9*), pUDE756 (*KmURA9*) and pUDE809 (*OpURA9*) yielded strains IME569, IME570, IME571, IME572 and IME573, respectively. Similarly, CEN.PK113-5D was transformed with pUDE1069 (*ScURA1*) resulting in IME603. After plating on SMUD without uracil, plasmid identity was verified by diagnostic PCRs with oligonucleotide 2989, which binds in the plasmid backbone, and either oligonucleotide 15597, 14875, 16454, 16443, 16451 or 12735, which bind within *Arura9, DbURA9, SjURA9, KmURA9,* *OpURA9* or *ScURA1*, respectively.

Strains IME600, IME601, IME602 and IME604 were constructed from IMK976 (*leu2Δ ura1Δ*) by co-transformation of pUDC286 (*COX4-MTS-mRuby2*) and a plasmid expressing a *URA9* gene fused to eGFP. Transformation with pUDE849 (*Arura9-eGFP*), pUDE1008 (*SjURA9-eGFP*), pUDE1009 (*DbURA9-eGFP*) and pUDE1011 (*OpURA9-eGFP*) and selection on SMUD without uracil and leucine yielded strains IME600, IME601, IME602 and IME604, respectively. Presence of pUDC286 was verified with primers 14345/1552. Presence of plasmids pUDE849, pUDE1008, pUDE1009 and pUDE1011 was confirmed by PCRs with primer 15651 combined with primer 15598, 14480, 16460 or 15645, respectively.

To introduce single-nucleotide mutations in *Arura9*, *SjURA9* and *KmURA9*, the codon of interest (encoding either a serine or a cysteine) was replaced by a *kanM*X marker in a first round of transformation, after which, in the resulting strain, the marker was again replaced by a fragment harboring the mutated codon in a second step (Additional file 3; Figure S4 [5]. The *kanMX* marker cassettes flanked by *Arura9, SjURA9* and *KmURA9* sequences were constructed by PCR, using pROS13 as template and oligonucleotide pairs 15851/15852, 15847/15848 and 15837/15838, respectively. Strains IMI432, IMI452 and IMI446 were then transformed with pUDR499, pUDR501 or pUDR602, targeting *Arura9*, *SjURA9* or *KmURA9* and the corresponding *kanMX* cassettes resulting in strains IMX2209, IMX2165 and IMX2203, respectively. After selection on YPD with G418, correct integration was verified by diagnostic PCR. Correct integration of the *kanMX* cassette was checked with primer 14760, which binds outside the *URA1* locus, and primer 1935, which binds inside the *kanMX* sequence. The integrated *kanMX* markers were targeted in a subsequent transformation with pUDR605. Repair cassettes for strains IMX2209, IMX2165 and IMX2203 were constructed by annealing oligonucleotide pairs 15834/15835, 16370/16371 and 16368/16369, which harbored the desired single-nucleotide mutations in *Arura9*, *SjURA9* and *DbURA9*, respectively. Co-transformation of pUDR605 with the corresponding repair fragment into strains IMX2209, IMX2165 and IMX2203 yielded strains IMG007 (*Arura9*^C168S^), IMG005 (*KmURA9*^S263C^) and IMG008 (*SjURA9*^C265S^). Single colony isolates were selected on YPD with hygromycin and checked for correct integration by PCR using primer pair 11338/11353, which bind outside the *URA1* locus. Plasmids were removed by three sequential transfers on YPD, followed by plating on YPD and selecting single colonies that grew on YPD but not on YPD with hygromycin or geneticin. To confirm the presence of the single-nucleotide mutations, the *URA9* sequences of strains IMG007, IMG008 and IMG005 were amplified by PCR using primers 14759/14760, and purified products were Sanger sequenced (BaseClear B.V., Leiden, The Netherlands). *URA9* alleles in strains IMG007, IMG008 and IMG005 were Sanger sequenced using primers 12665, 14759, 14760 and 14872 binding outside the *URA9* gene. For sequencing of *Arura9*, *SjURA9* or *KmURA9* the additional oligonucleotides 14873/14874/14877/14878, 16439/16441/16443/16445 or 16438/16455/16456/16458 were used. Reads were assembled in Clone Manager Professional 9 (Sci-Ed Software, Westminster, CO, USA) using default settings for “Simple” assembly and aligned with the sequence of the corresponding non-mutated *URA9* allele.

Strain IMS1206 was obtained by three subsequent transfers of IME571 (*ura1* null mutant expressing *SjaURA9* from a plasmid), on non-selective media (SMUD-ura). The culture was plated on SMD-ura and a single colony isolate were re-streaked on SMD and SMD-ura. Loss of pUDE815 (*SjURA9*) was confirmed by the inability to grow on medium without uracil. Subsequent selection of a single colony isolate by three subsequent restreaks on SMUD yielded strain IMS1206.

Strains IMS1167, IMS1168, IMS1169 and IMS1170 were obtained from two anaerobically grown cultures of strain IMI439 (*DbURA9*) on SMUD. To avoid carry-over from an aerobic environment, cultures were plated on SMD within the anaerobic chamber. Two single colony isolates per plate were re-streaked (three times) anaerobically, resulting in strains IMS1167, IMS1168, IMS1169 and IMS1170.

### References

1. Raab D, Graf M, Notka F, Schödl T, Wagner R. The GeneOptimizer Algorithm: Using a sliding window approach to cope with the vast sequence space in multiparameter DNA sequence optimization. Syst Synth Biol. 2010;4:215–25.

2. Lee ME, DeLoache WC, Cervantes B, Dueber JE. A highly characterized yeast toolkit for modular, multipart assembly. ACS Synth Biol. 2015;4:975–86.

3. Mans R, van Rossum HM, Wijsman M, Backx A, Kuijpers NGA, van den Broek M, Daran-Lapujade P, Pronk JT, van Maris AJA, Daran J-MG. CRISPR/Cas9: a molecular Swiss army knife for simultaneous introduction of multiple genetic modifications in *Saccharomyces cerevisiae*. FEMS Yeast Res. 2015;15:fov004.

4. Bouwknegt J, Wiersma SJ, Ortiz-Merino RA, Doornenbal ESR, Buitenhuis P, Giera M, Müller C, Pronk JT. A squalene–hopene cyclase in *Schizosaccharomyces japonicus* represents a eukaryotic adaptation to sterol-limited anaerobic environments. Proc Natl Acad Sci. 2021;118:e2105225118.

5. Beekwilder J, van Rossum HM, Koopman F, Sonntag F, Buchhaupt M, Schrader J, Hall RD, Bosch D, Pronk JT, van Maris AJA, Daran JM. Polycistronic expression of a β-carotene biosynthetic pathway in *Saccharomyces cerevisiae* coupled to β-ionone production. J Biotechnol. Elsevier B.V.; 2014;192:383–92.
